# Domain organization of DNase from *Thioalkalivibrio* sp. provides insights into retention of activity in high salt environments

**DOI:** 10.3389/fmicb.2015.00661

**Published:** 2015-07-01

**Authors:** Gediminas Alzbutas, Milda Kaniusaite, Algirdas Grybauskas, Arunas Lagunavicius

**Affiliations:** ^1^Department of Eukaryote Gene Engineering, VU Institute of Biotechnology, Vilnius UniversityVilnius, Lithuania; ^2^Thermo Fisher ScientificVilnius, Lithuania; ^3^Faculty of Chemistry, Vilnius UniversityVilnius, Lithuania

**Keywords:** DNase, *Thioalkalivibrio* sp., HhH motif, DNA binding domain, halophile, adaptation

## Abstract

Our study indicates that DNA binding domains are common in many halophilic or halotolerant bacterial DNases and they are potential activators of enzymatic activity at high ionic strength. Usually, proteins adapt to high ionic strength by increasing the number of negatively charged residues on the surface. However, in DNases such adaptation would hinder the binding to negatively charged DNA, a step critical for catalysis. In our study we demonstrate how evolution has solved this dilemma by engaging the DNA binding domain. We propose a mechanism, which enables the enzyme activity at salt concentrations as high as 4 M of sodium chloride, based on collected experimental data and domain structure analysis of a secreted bacterial DNase from the extremely halotolerant bacterium *Thioalkalivibrio* sp. *K90mix*. The enzyme harbors two domains: an N-terminal domain, that exhibits DNase activity, and a C-terminal domain, comprising a duplicate DNA binding helix-hairpin-helix motif. Here we present experimental data demonstrating that the C-terminal domain is responsible for the enzyme's resistance to high ionic strength.

## 1. Introduction

Eukaryotic DNaseI is commonly used to clear DNA contamination from RNA samples, however, it is very salt-sensitive. Over the course of evolution the DNases of halophilic/halotolerant organisms might have adapted to work at high salt concentration. Several nucleases resistant to ionic strength were discovered in the past decades (Kamekura and Onishi, 1974, 1978; Onishi et al., 1983; Kanlayakrit et al., 2001). However, these studies didn't reveal any mechanism of halotolerance.

In 1998 Pan and Lazarus published their attempts to design an eukaryotic DNaseI, which would retain its activity at the elevated ionic strength (Pan and Lazarus, 1998). Since high ionic strength hinders the interaction between the enzyme and the DNA, the authors tackled this problem by introducing additional positive residues onto the DNA-binding surface of the nuclease catalytic domain (Pan and Lazarus, 1998). Meanwhile, our analysis of the sequence data from the halophilic/halotolerant prokaryotes indicates that evolution used a completely different approach: the interaction between the enzyme and the substrate is stabilized by an additional C-terminal DNA-binding domain within the enzyme. We have discovered that many DNases from halophilic/halotolerant species are multi domain proteins. This fact led to the hypothesis that in some cases a fusion of an additional domain to the DNase domain was the key factor in evolution, which enabled the activity of bacterial DNases at high ionic strength. To test this hypothesis, we selected a DNase with a DNA binding domain, in addition to a nuclease domain, from an extremely halotolerant bacterium, *Thioalkalivibrio* sp. *K90mix*, for experimental analysis. Along with the recombinant (hexahistidine-tagged) enzyme we also analyzed its two mutants: one lacking the C-terminal domain and the other one harboring an inactivating mutation in the active site of the DNase domain. In this article we present halophilic properties of the DNase from *Thioalkalivibrio* sp. *K90mix* and its salt tolerance strategy. It has been shown that accumulation of negatively charged acidic surface residues is related to adaptation for high salt concentrations (Siglioccolo et al., 2011; Graziano and Merlino, 2014). Thus, this evolutionary adaptation could potentially lower the enzyme's affinity to negatively charged DNA. Here we present an example, where accumulation of negatively charged residues on the surface of bacterial DNase is accompanied and potentially alleviated by a DNA binding domain.

## 2. Materials and methods

### 2.1. Analysis of microbial DNaseI family proteins and the resistance of corresponding micro-organisms to salt

Initially, IPR016202 protein family sequences were collected from the InterPro database (accessed in June, 2014) (Hunter et al., 2012). The sequences were matched to UniRef90 clusters (UniProt Consortium, 2014) and subsequent analysis was performed on representative sequences from these clusters. All non-prokaryotic sequences were discarded. Remaining sequences were subjected to phylogenetic analysis and domain detection. A maximal molar NaCl concentration allowing growth of a corresponding organism was inferred for each analyzed sequence. In some cases, the maximal NaCl concentration value was found in the published data, in other cases, the salt tolerance was inferred based on a living environment or a cultivation medium. Six arbitrary selected salt tolerance categories were used. The first category, where the maximum salinity is indicated as “close to 0” encompasses the organisms that were not considered in the literature as being salt tolerant or their living environment/growth medium does not imply salt tolerance. The second one (“<0.8”) encompasses slightly halophilic/halotolerant species. An organism was assigned to this category if the corresponding concentration of NaCl was explicitly indicated in the literature or the microorganism was collected from marine habitats. The assignments to the other four categories (“0.8–0.9,” “1.0–1.4,” “1.5–2.0,” “3.4–5.1”) corresponding to medium-extreme halophilic/halotolerant species were based on the explicit statements in the literature.

Domains in the sequences of bacterial DNases were detected using InterProScan 5.4-47.0 (Jones et al., 2014). Phylogeny analysis of the corresponding sequences was performed using Phyrn-1.7.2 package (Bhardwaj et al., 2012). Five thousand replicates of the distance matrices were generated for bootstrapping. The corresponding neighbor-joining trees were calculated and a consensus tree was produced using the NEIGHBOR and CONSENS programs from the PHYLIP package (Felsenstein, 1989). ETE 2.2 package was used for the visualization of the tree and supplementary information (Huerta-Cepas et al., 2010).

A secretion signal search was performed in the sequences of DNases from organisms, that can grow in 1.5 M or higher NaCl concentrations. A secretion signal was detected using three programs: (1) SignalIP 4.1 (Petersen et al., 2011) was used for gram-negative bacteria sequences; (2) PRED-SIGNAL (Bagos et al., 2009) was used for an archaeal sequence (*Methanohalobium evestigatum*) ; (3) Philius (Reynolds et al., 2008) was used for the prediction of the presence of a secretion signal and protein type.

### 2.2. Cloning, expression, and purification of proteins

Gene of the recombinant DNase from *Thioalkalivibrio* sp. *K90mix* (DNaseTA) was *de novo* synthesized by DNA 2.0 (California, USA) and codons were optimized for expression in *E. coli*. Protein sequence was taken from an Uniprot entry (accession code D3SGB1), the secretion signal sequence was excluded. The gene was cloned into a pLATE31 vector (Thermo Fisher Scientific, #K1261). The coding sequence of the C-terminal His_6_-tag originated from the vector.

Two mutants of the his-tagged DNaseTA were constructed: an active site mutant with the inactivating mutation [H134A (Chen et al., 2007)], denoted as DNaseTA H134A and a mutant with the removed C-terminal domain denoted as DNaseTA ΔC. The coding sequence of the C-terminal His_6_-tag originated from the vector and was added during cloning into a pLATE31 vector (Thermo Fisher Scientific, #K1261). DNaseTA H134A was generated by two-steps megaprimer PCR. Both PCR reactions were performed with 2x Phusion High Fidelity PCR Master Mix (Thermo Fisher Scientific, #F-548S). Primer sequences for both PCR steps are shown in Supplementary Material (Table S1). DNaseTA ΔC mutant was constructed via single PCR step, the primer sequences are shown in Supplementary Material (Table S1). The PCR products were cloned into a pLATE31 vector (Thermo Fisher Scientific, #K1261). The coding sequences of DNaseTA, DNaseTA H134A, DNaseTA ΔC are shown in Supplementary Material Figure S1.

As a reference enzyme, his-tagged bovine DNaseI was cloned into a pLATE51 vector (Thermo Fisher Scientific, #K1271). The DNA sequence corresponding to the N-terminal His_6_-tag originated from the vector and was added during cloning.

The constructs were cloned using *E. coli* ER2267 strain (New England Biolabs). The bacteria were grown on LB agar supplemented with 2% (w/v) glucose and carbenicillin. For the protein expression the constructed recombinant plasmids were transformed into *E. coli* ER2566 strain (New England Biolabs). Bacteria were grown in LB broth supplemented with glucose 1% (w/v) and carbenicillin (50 μg/ml). A preculture was grown till OD_600_ 0.3 at 37°C and then used for inoculation. A main culture was inoculated with 1/40 of the preculture and grown at 37° C until the induction. The expression was induced by addition of IPTG (1 mM) when OD_600_ reached 0.8–0.9 and subsequently grown at 23° C for 16 h. Before the induction the culture was cooled on ice. Bacteria were lysed chemically and expressed proteins were purified in one step using nickel affinity spin columns (Thermo Fisher Scientific, #88225). The washing buffer had following composition: 2% Triton X-100; 20 mM Tris-HCl, pH 7.5; 0.5 M NaCl; 5 mM CaCl_2_; 20 mM imidazole, pH 7.8. The elution buffer had following composition: 2% Triton X-100; 20 mM Tris-HCl, pH 7.5; 0.5 M NaCl; 5 mM CaCl_2_; 250 mM imidazole, pH 7.8. The eluates were dialysed against buffer having following composition: 50 mM Tris-acetate, pH 7.5; 10 mM CaCl_2_; 50% glycerol. When requirements for divalent cations were analyzed, 0.5 M EDTA solution was added to the eluates to get 100 mM EDTA concentration and then the eluates were dialyzed against buffer having following composition: 50 mM Tris-acetate, pH 7.5; 10 mM EDTA; 50% glycerol. The concentration of the purified enzymes was assessed by SDS-PAGE and subsequent densitometry.

### 2.3. Activity assays

#### 2.3.1. Digestion of long DNA substrate

Two micro gram pUC19 DNA cleaved with SmaI was digested with 2.5 nM of enzyme. Two ranges of NaCl concentration were explored: 0–1 M in 0.1 M increments and 0–4 M in 0.4 M increments. The reactions were performed for 10 min at 37°C in 100 μl of the reaction mixture with 10 mM Tris-HCl, pH 7.5 and varying amount of CaCl_2_ and MgCl_2_. Thermo Scientific ZipRuler Express DNA Ladder 2 (#SM1373) was used as a molecular weight standard to evaluate the degradation of the DNA substrate.

#### 2.3.2. Digestion of short DNA substrate

Ten nano meter 16 bp DNA (2 nM were labeled with ^33^P at 5'-end) was digested with 0.66 nmol of enzyme at 37°C in 100 μl of a reaction buffer: 10 mM Tris-HCl, pH 7.5; 10 mM CaCl_2_; 10 mM MgCl_2_. 9 μl of the reaction mixtures were removed at 1, 2, 4, 8, 16, 32, 64, 128, 192 min after start. The samples were mixed with 9 μl of 2x RNA loading dye (Thermo Fisher Scientific, #SM1373), heated for 5 min at 95°C and analyzed by denaturing PAGE. The half-life of the substrate digestion was estimated during subsequent densitometry.

### 2.4. Modeling and analysis of DNase domain

The protein sequence of DNaseTA was downloaded from the corresponding entry in UniproKB database (UniProt Consortium, 2014) (http://www.uniprot.org/uniprot/D3SGB1). HHblits (Remmert et al., 2012) from HH-suite 2.0 was used to identify the protein domains in DNaseTA. The modeling of the DNase domain was performed using I-Tasser server (Roy et al., 2010). The models were refined with Kobamin (Rodrigues et al., 2012), a knowledge-based potential refinement program. The best quality model was selected using Prosa-web (Wiederstein and Sippl, 2007). Prosa-web was used for backbone and Qmean (Benkert et al., 2008) for side chain quality assessment. The electrostatic surface potential of DNase domain of DNaseTA was calculated with APBS tools (Baker et al., 2001) and visualized in Pymol (Schrödinger, unpublished). The structure was prepared for electrostatics calculations by adding hydrogens with PDB2PQR (Dolinsky et al., 2004) using Amber force field (Case et al., 2014). The range from −5 kT/e in red to +5 kT/e in blue was chosen for surface coloring. The model for a DNase from *Methanohalobium evestigatum* was created and analyzed in a similar manner. For evaluation of the surface residues conservation level proteins homologous to DNaseTA were identified using PSI-Blast (Altschul et al., 1997) and Jackhammer (Eddy, 2011), profile-profile alignment search tools. Homologous proteins were aligned using multiple alignment program MAFFT (Katoh and Standley, 2013) with L-INS-i (Katoh and Toh, 2008) option. Lastly, multiple-aligned sequences were imported into a Consurf (Celniker et al., 2013) program for mapping of conserved amino acids. In order to indicate potential ion and DNA binding residues in DNaseTA, the DNase domain of DNaseTA, DNaseI structures PDB ID: 4AWN, 2A3Z, 3DNI (known positions of ions) and PDB ID: 1DNK (known positions of DNA) were superimposed. The superimposition was performed using Dali Server (Holm and Rosenström, 2010) and mapped in Jalview Waterhouse et al. (Holm and Rosenström, 2010).

## 3. Results

### 3.1. DNaseI family sequences properties and halotolerance of corresponding micro-organisms

**Figure 1:**
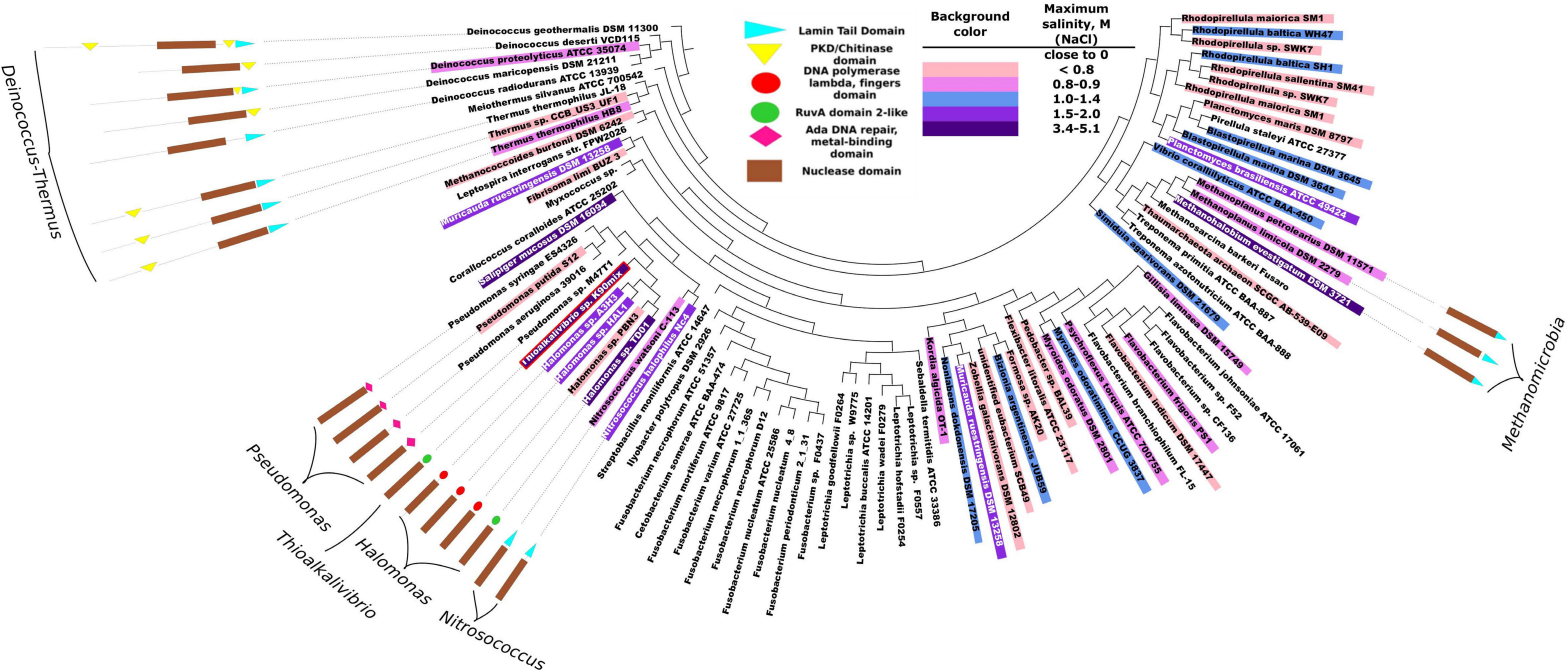
**Phylogeny analysis of bacterial proteins of DNaseI family and their domain organizations**. Each leaf corresponds to one UniRef90 cluster (group of sequences with at least 90% pairwise identity). The corresponding accession codes are given in Supplementary Material (Table S2). The domain structure is presented only if the representative protein of the cluster has other domains in addition to the nuclease domain. Background colors indicate an inferred maximum concentration of NaCl at which the corresponding microorganism still grows. The more blue background – the more salt tolerant the microorganism is. DNA polymerase lambda fingers domain and RuvA domain 2-like domain are similar (red and green circles) as both correspond to duplicate HhH (helix-hairpin-helix) motifs. Red border indicates a DNase from *Thioalkalivibrio* sp. *K90mix* which was selected for experimental analysis.

The purpose of phylogenetic and domain structure analysis of bacterial DNases was to identify the domains, which would be potentially related to halotolerance or halophilicity. The analysis of the InterPro 4.7 database (Hunter et al., 2012) revealed that there are about 300 prokaryotic proteins belonging to DNaseI family. Clustering these sequences at 90% sequence identity level resulted in 86 clusters (Uniref90). Manual inspection of available data in literature revealed that more than a half of the representative sequences originate from halophilic/halotolerant organisms. The accession codes of the analyzed DNaseI family proteins, corresponding organisms and collected data on salt tolerance are in Supplementary Material (Table S2). The summary of this data is given in Figure 1. In this figure species corresponding to 86 representative sequences are indicated along with the inferred maximum salinities (concentration of NaCl) at which microbial growth occurs. In total we have collected data on 82 prokaryotic organisms. 39 of them were considered to be of low salt tolerance, 16 were classified as being slightly halophilic/halotolerant (≥0.3 and <0.8 M NaCl), 23 were classified as being medium halophilic/halotolerant (≥0.8 and <3.4 M NaCl) and 4 species were classified as extremely halophilic/halotolerant (≥3.4 M NaCl).

The phylogeny of the sequence fragments, which correspond to the nuclease domain of the proteins, was analysed and the topography of the resulting phylogeny tree is given in Figure 1. All the domain names used in the figure are the same as used in InterPro 4.7 database, except for the nuclease domain. We named exo/endo/phospho and DNase domain (note that all analyzed proteins belong to DNaseI family) as nuclease domain. Our analysis indicates that bacterial DNases apart from their nuclease domain might have 5 types of other domains: Lamin tail, PKD/Chitinase, Ada DNA repair/metal binding, RuvA domain2-like and DNA polymerase lambda fingers. The last two domains: the RuvA domain 2-like (Singh et al., 2002) and the DNA polymerase lambda fingers (Garcia-Diaz et al., 2004) are similar (shown as red and green circles in Figure 1) as both of them correspond to the duplicate HhH (helix-hairpin-helix) motifs (Hunter et al., 2012). Domain analysis revealed that the DNases of *Deinococcus-Thermus* phylum stand out as a distinctive group: the proteins are longer compared to other bacterial DNases and contain significant sequence fragments that cannot be assigned to any functional domain. These proteins have either PKD/Chitinase or Lamin tail domain, or both. We examined the potential relationship between the existence of the detected domains (except for the DNase domain) and the resistance to ionic strength. All the subsequent references to salt tolerance are based on this figure. The corresponding literature sources are indicated in Supplementary Material (Table S2).

If a protein is secreted, then it should be adapted to the surrounding environment. Thus additional search for secretion signal was performed on the sequences originating from the organisms which can grow at concentration of NaCl ≥1.5 M. The results are given in Table 1.

**Table 1:** **The secretion signal in DNases from the most salt tolerant organisms (≥slant1.5 M NaCl)**.

**Representative sequence**	**Representative species/label used in phylogeny tree**	**Secretion signal**		**Protein type prediction by Philius**		**Additional domain**	**Maximum NaCl, M**
		SignalIP 4.1^*a^	PRED-SIGNAL^*a^	**Type**	**Confidence**		
D7E828	*Methanohalobium evestigatum DSM 3721*	-	Y	Globular with signal peptide	0.99	Lamin tail	5.1^a^
D3SGB1	*Thioalkalivibrio* sp. *K90mix*	Y	-	Globular with signal peptide	0.99	duplicate HhH^*b^	4^b^
F7SPZ3	*Halomonas* sp. *TD01*	Y	-	Globular with signal peptide	0.98	duplicate HhH^*b^	3.42^c^
S9QWK2	*Salipiger mucosus DSM 16094*	N	-	Globular with signal peptide	0.71	-	3.42^d^
G4F511	*Halomonas* sp. *HAL1*	Y	-	Globular with signal peptide	0.99	duplicate HhH^*b^	2^e^
F0SFA3	*Planctomyces brasiliensis ATCC 49424*	N	-	Transmembrane	0.68	-	1.72^f^
D8KCF8	*Nitrosococcus halophilus Nc4*	Y	-	Globular with signal peptide	0.99	Lamin tail	1.60^g^
G2PLU4	*Muricauda ruestringensis DSM 13258*	N	-	Globular	0.99	-	1.54^h^
G2PQ99	*Muricauda ruestringensis DSM 13258*	N	-	Globular with signal peptide	0.99	-	1.54^h^
T2LFY5	*Halomonas* sp. *A3H3*	N	-	Globular with signal peptide	0.91	duplicate HhH^*b^	1.5^i^

a *Wilharm et al., 1991*.

b *Muyzer et al., 2011*.

c *Tan et al., 2011*.

d *Martínez-Cánovas et al., 2004*.

e *Lin et al., 2012*.

f *Schlesner, 1989*.

g *Koops et al., 1990*.

h Bruns et al., 2001.

i *Koechler et al., 2013*.

*^a^ *Resulting decision on the existence (Y) or not existence (N) of the secretion signal sequence*.

*^b^ *Corresponds to RuvA domain 2-like domain or DNA polymerase lambda fingers domain*.

### 3.2. DNaseTA requirement of divalent ions and resistance to ionic strength

**Figure 2:**
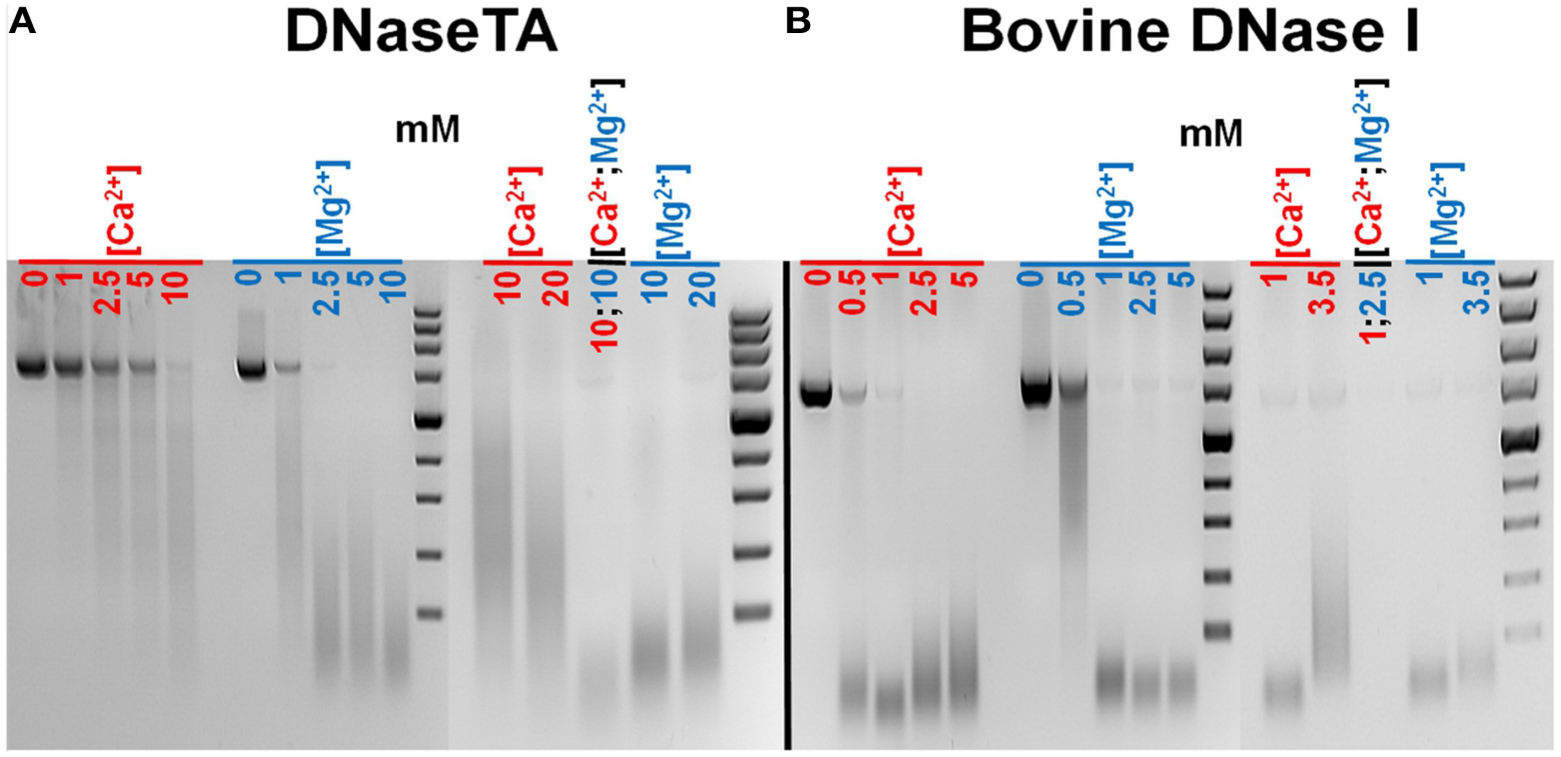
**Different requirements for divalent ions of DNase from *Thioalkalivibrio* sp. *K90mix* (DNaseTA) and bovine DNaseI**. Several different concentrations of Ca^2+^ and Mg^2+^ were screened evaluating approximate optimum for DNaseTA **(A)** and bovine DNaseI **(B)**. It was found that both enzymes require Ca^2+^ and Mg^2+^ for activity, however, DNaseTA requires significantly higher concentrations. The optimal concentrations are ~10 mM Ca^2+^ and ~10 mM Mg^2+^ for DNaseTA and, respectively, ~1 mM and ~2.5 mM for bovine DNaseI.

We have estimated approximate optimal concentrations of divalent cations for the bovine DNaseI (a reference enzyme) and the DNase from *Thioalkalivibrio* sp. *K90mix* (DNaseTA). We observed (Figure 2) that both enzymes require Ca^2+^ or Mg^2+^ for catalytic activity and the maximum activity is achieved when both of these ion species are present. Data shows that DNaseTA requires significantly higher concentrations of divalent ions than bovine DNaseI: ~10 mM Ca^2+^ and ~10 mM Mg^2+^ is the optimal combination for the DNaseTA (Figure 2A), while for the bovine DNaseI the respective concentrations are ~1 mM and ~2.5 mM (Figure 2B).

We have analyzed the influence of increasing salt (NaCl) concentration on DNA hydrolysis by DNaseTA and its two mutants. We assayed digestion of two DNA types: (i) long plasmid, (ii) short duplex. This was done in order to double-check the findings as the data on the digestion of the long plasmid were qualitative agarose gel images and it was accompanied by quantitative data on the short substrate digestion.

**Figure 3:**
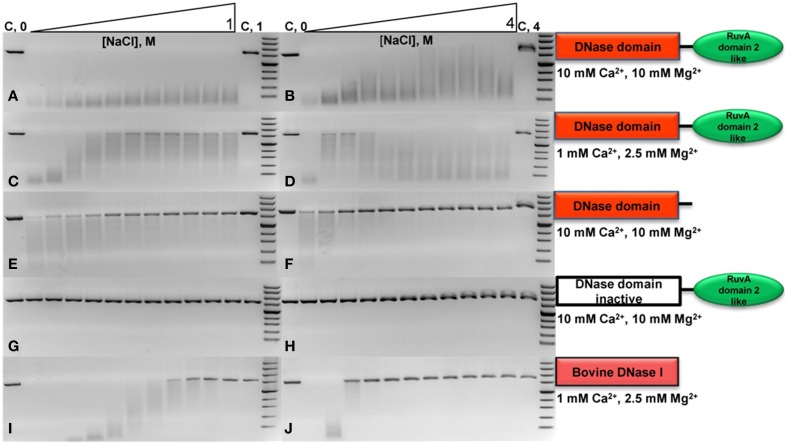
**Activity of DNase from *Thioalkalivibrio* sp. *K90mix* (DNaseTA), its mutants and bovine DNaseI at different ionic strengths**. Activity of analyzed proteins was evaluated by digestion of linearized pUC19 plasmid in the presence of various concentrations of NaCl. Two series of NaCl gradients were used: the first one corresponds to a range from 0 to 1 M with increments of 0.1 M (left electrophoregrams), the second one corresponds to a range from 0 to 4 M with increments of 0.4 M (right electrophoregrams). Activity assays in case of DNaseTA were performed comparing two compositions of divalent ions: a suboptimal one, corresponding to lower **(C,D)** concentrations, and near optimum one, which corresponds to higher concentrations **(A,B)**. The data on the mutant with the removed C-terminal domain (DNaseTA ΔC) is given at the **(E,F)** panels. The data on DNaseTA with mutation at DNase active site (DNaseTA H134A) is given at the **(G,H)** panels. Analogous data for Bovine DNaseI is given at the **(I,J)** panels (the near optimum concentration of divalent cations for DNaseI were used).

The digestion of the long substrate by the DNaseTA was assayed under two conditions in terms of divalent cations : (1) the near optimum combination of Ca^2+^ and Mg^2+^ concentrations for DNaseTA (Figures 3A,B). (2) the lower concentrations of these divalent ions, which resembles near optimum conditions for the bovine DNaseI (Figures 3C,D). The results indicate that DNaseTA digests DNA in the presence of high salt concentration (up to 4 M NaCl) at both concentrations of divalent ions, however, apparent differences between those two combinations are noticeable at lower ionic strength (up to ~1 M NaCl). DNaseTA digests DNA at the lower ionic strength and higher concentration of divalent ions, although the length of final product gradually increase (presumably due to decreasing DNase activity). Contrastingly, under lower concentration of divalent ions the ability of DNaseTA to digest DNA decreases significantly when salt concentration reaches ~0.4 M NaCl. At this point some substrate remains even undigested. Further increase in ionic strength, however, reinforces the degradation of DNA by DNaseTA as the length of the DNA substrate is shortened significantly even at 4 M NaCl.

The digestion of the long substrate by the mutants of DNaseTA were analyzed at the near optimum combination of divalent ions for DNaseTA. The truncated form of DNaseTA with the removed C-terminal domain (DNaseTA ΔC) retains its DNase activity as presented in Figures 3E,F. This mutant of DNaseTA noticeably digest the DNA substrate only up to ~0.6 M NaCl and the DNA digestion is completely inhibited at NaCl concentrations higher than 1.2 M. The mutant of DNaseTA, which has the inactivating mutation H134A, (DNaseTA H134A) is unable to digest DNA at any of the tested concentrations of NaCl (Figures 3G,H).

The data corresponding to the bovine DNaseI is given in Figures 3I,J, which indicates that the concentration of NaCl above ~0.9 M totally inhibits DNA digestion. In this case near optimum combination of divalent ions for the bovine DNaseI were used.

The data on the digestion of the short DNA substrate is presented in the Table 2. In this experiment for analysis of DNaseTA and its mutants the near optimum combination of divalent ions for DNaseTA was used. For the analysis of DNaseI—the corresponding near optimum concentration of divalent cations was used. It complements the data on the long substrate digestion and indicates that DNaseTA is able to digest DNA in the presence of 4 M NaCl, the truncated version of the DNaseTA (without C-terminal domain) is active only at low ionic strength, and the DNaseTA active site mutant is inactive at any ionic strength.

**Table 2:** **The half-life of the radioactive substrate digestion of the recombinant DNase from *Thioalkalivibrio* sp. *K90mix* (DNaseTA) and its mutants**.

**NaCl, M**	**DNaseTA**	**DNaseTA** **ΔC**	**DNaseTA H134A**
	**Half-life of substrate digestion T_1/2_, min**		
0	9.43	31.29	ND^a^
0.5	43.39	ND^a^	ND^a^
1.2	60.18	ND^a^	ND^a^
4.0	47.66	ND^a^	ND^a^

a *ND, not detectable*.

### 3.3. Surface properties of dnase domain of DNaseTA

**Figure 4:**
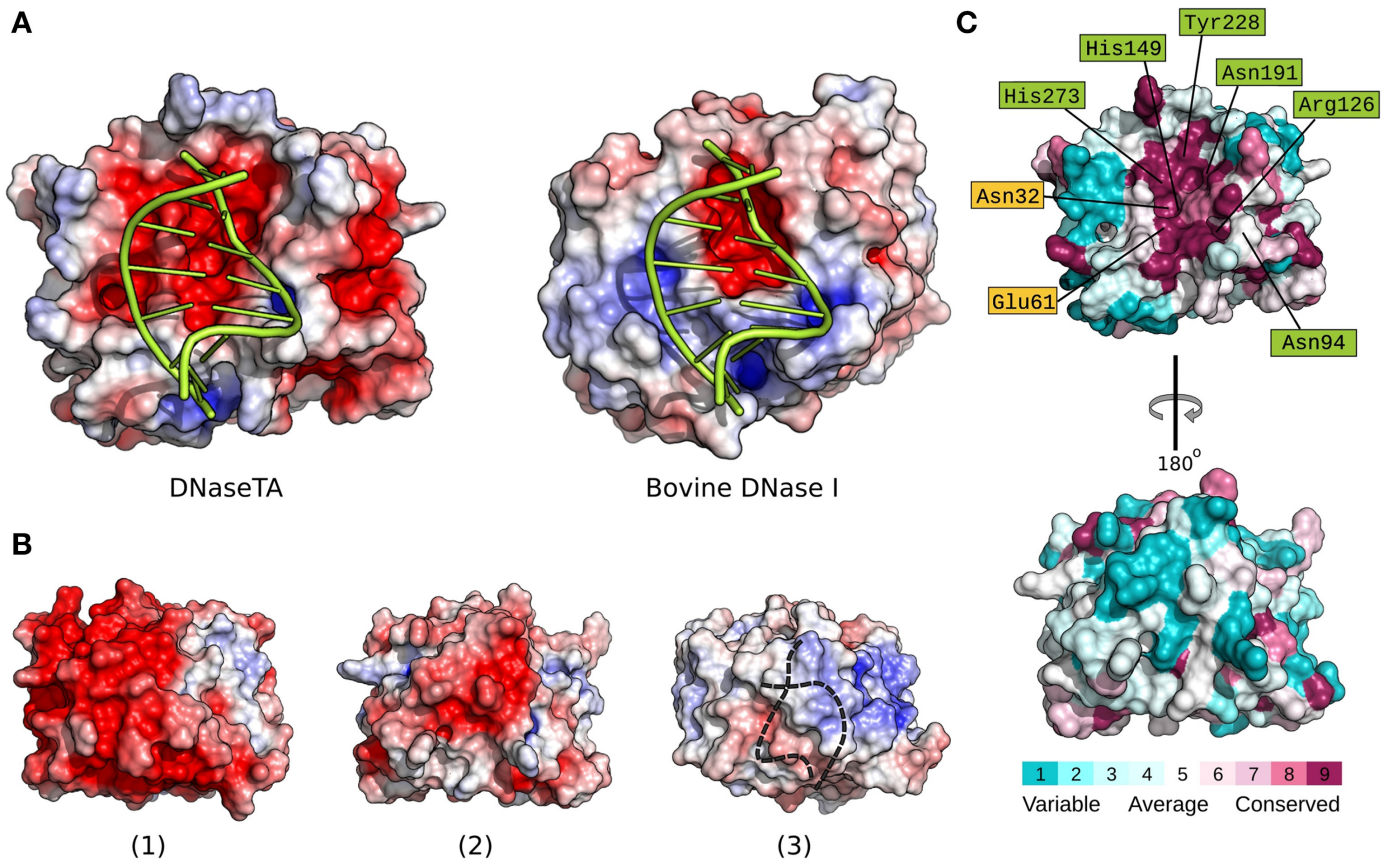
**Electrostatic surface potential and evolutionary conserved residue maps**. Difference between DNase domain of DNase from *Thioalkalivibrio* sp. *K90mix* (DNaseTA) and bovine DNaseI electrostatic potential surfaces, which are in contact with DNA, are depicted in **(A)**. Here DNA is positioned based on crystallographic bovine DNaseI structure (PDB ID: 1DNK). Electrostatic surface potential maps of DNase domains' sides which are not facing DNA are depicted in **(B)**: DNase from *Methanohalobium evestigatum* (DNaseME) **(1)**, DNaseTA **(2)**, and bovine DNaseI **(3)**. Dashed line in **(3)** represents DNA position behind DNaseI. Evolutionary conserved residue map of DNase domain model of DNaseTA is depicted in **(C)**. The front (top structure) that faces DNA, and the back (bottom structure) sides of the proteins are visualized. Scales from variable (cyan) to conserved (purple) are shown under the structures.

In order to elucidate the apparent differences we find between DNaseTA and bovine DNaseI regarding the ability to digest DNA at high ionic strength, we analyzed the electrostatic potential properties and conservation of surface residues of the DNase domain. The active site residues, which interact with the DNA and are involved in catalysis, are marked in green color and the residues, which bind magnesium ions, are marked in yellow color in Figure 4C and Figure S2 (Supplementary Material). As we see in Figure 4 an electrostatic potential (the redder—the more electronegative) of the surfaces indicates that the active site regions of DNaseTA and bovine DNaseI are both negatively charged. However, the DNaseTA has significantly larger electronegative patch in the DNA binding surface than the bovine DNaseI (Figure 4A). The fact that the predicted surface of DNaseTA is more electronegative compared to DNaseI is also evident when we compared those parts of protein surfaces which are not facing DNA [Figures 4B(2),B(3)]. Therefore, the surface of DNaseTA is overall more electronegative than the surface of bovine DNaseI. For comparison, along with DNaseTA and bovine DNase in Figure 4B(1) we visualized the surface electrostatic potential of the DNase from *Methanohalobium evestigatum* (DNaseME). *Methanohalobium evestigatum* is the most salt tolerant organism, which was included in our research. The maps demonstrate that the DNaseME has the most electronegative surface out of all three DNases. In Figure 4B a protein from the most halophilic/halotolerant microorganism is on the left (1 – DNaseME), a protein from less halophilic/halotolerant organism is in the middle (2 – DNaseTA) and an eukaryotic protein is on the right (3 – bovine DNaseI). For the references on the salt tolerance please see the Table 1. Thus the more halophilic the DNase is, the more electronegative surface it has.

Alongside with the electrostatic potential we also mapped the conservation of the DNaseTA surface residues (Figure 4C). The results show that the positions of the active site and DNA binding residues Asn94, Arg126, His149, Asn191, Tyr228, His273 along with the Mg^2+^ binding residues Asn32, Glu61 are conserved, while many other surface residues are variable. It is evident that the surface side which is not facing DNA is variable and does not show any strong evolutionary conservation (see Figure 4C bottom surface). Therefore, the surface residues in the homologs of DNaseTA are mostly variable and thus give room for evolutionary adaptations.

## 4. Discussion

In this study we analyzed the data on the phylogeny of microbial DNases, their domain structure and salt tolerance of the corresponding microorganism (Figure 1). We hypothesized that the additional DNA binding domains might serve for the adaptation of the microbial DNases to saline environments. Our studies have confirmed the existence of additional domains in the prokaryotic, potentially salt tolerant DNases. The analysis revealed that two types of domain organizations are found exclusively in DNases from halophilic/halotolerant species: (i) the C-terminus of DNase fused to a Lamin tail domain, (ii) the C-terminus of DNase fused to a domain containing HhH duplicate motifs (RuvA domain2-like or DNA polymerase lambda fingers). These two types of motifs are not homologous, however, they could be analogous in function. The HhH duplicate domain binds to DNA and is found in many DNA interacting proteins (Doherty et al., 1996). The Lamin tail domain was discovered in nucleus envelope of the eukaryotic cells and is also associated with the binding to DNA (Mans et al., 2004; Bruston et al., 2010). As it is well established, ionic strength has a diminishing effect on electrostatic interactions. Therefore, an additional DNA binding domain should enhance the activity of DNases in the presence of the elevated ionic strength.

We propose that in order for a microbial DNase to hydrolyse DNA in the presence of elevated ionic strength, it should have a DNA binding domain. Amongst organisms, that can tolerate salinity of ~1.5 M and more (Figure 1), we discovered nine organisms and ten DNases. Out of the nine organisms eight were bacteria. For adaptation to high salinity bacteria usually employ “salt-out” strategy using compatible solutes (da Costa et al., 1998) and their intracellular proteins might not be adapted to high salt concentrations. However, if proteins from halotolerant bacteria have secretion signals then, most likely, they enter a secretory pathway and, if are secreted, they should be evolutionary adapted to elevated ionic strength. Therefore, we have searched for the presence of a secretion signal (Table 1) and have found that only five of the ten DNases have a secretion signal, which is detectable by two programs that were used in this study. These five DNases have other domains in addition to the nuclease domain. Three of them have a domain with duplicate HhH motifs that is fused to a nuclease domain, the other two proteins have a Lamin tail domain instead of the duplicate HhH motifs. Therefore we assumed that a DNase, which is potentially secreted via classical secretory pathway by a moderate-extreme halophile, should have an additional DNA binding domain. Thus, for our investigations we have selected a DNase, which has a C-terminal domain with duplicate HhH motifs, and examined if this domain is at least partially responsible for the resistance to high ionic strength. The chosen DNase is from an extremely halotolerant species *Thioalkalivibrio* sp. *K90mix*.

The first task in performing experimental assays with this enzyme was to evaluate the need for divalent cations. The data (Figure 2) indicates that higher concentrations of divalent ions are required for the optimal activity of DNaseTA compared to its bovine counterpart. The analysis of the bovine DNase (Guéroult et al., 2010) showed that the divalent ions are essential for DNA binding, as without bound ions the DNA binding surface in eukaryotic DNase is electronegative and thus unable to bind DNA. The electrostatic DNA binding surface potential of the bovine DNaseI and the DNase from *Thioalkalivibrio* sp. *K90mix* are visualized in Figure 4A. These data might explain why the prokaryotic DNase requires higher concentration of divalent ions. The DNA binding surface of the DNase from *Thioalkalivibrio* sp. *K90mix* has a much larger patch of electronegative area (Figure 4A). This implies that more divalent ions have to be bound to DNaseTA in order to make the surface electro-positive and enable the binding to DNA.

Further we explored the capability of DNase from *Thioalkalivibrio* sp. *K90mix* to digest DNA substrates in a series of buffers where NaCl concentration was gradually increased (Figure 3). In these assays two compositions of divalent ions were compared: one suboptimal, with lower concentrations, and the second, using near optimal concentrations. Interestingly, the concentration of divalent ions remarkably influences the resistance of the DNase to the ionic strength in buffers with NaCl concentration up to ~1 M. When the concentration of divalent ions was low, gradual increase in a salt concentration revealed two peaks of the activity: one in the absence of NaCl and the second, when the concentration of NaCl was above 1.2 M (Figures 3C,D). This observation was reproduced in several experiments (data not shown). In contrast, when the concentration of divalent ions was higher, there was no activity suppression by moderate concentrations of NaCl and a recovery by higher concentrations. This implies that at the concentrations of NaCl ~1.2 M and higher, divalent ions aid the enzyme to maintain its activity and resist to the increasing ionic strength. As increasing ionic strength weakens DNA protein interaction, our data suggests that at the higher concentration of divalent ions the prokaryotic DNase has higher affinity to DNA than at the lower concentrations.

It is still unclear why during the gradual increase of ionic strength at low concentration of divalent ions the dual activity peak was observed. However, this could be explained by presuming that the C-terminal domain of the prokaryotic DNase acts in its full potential only at high salt concentrations and it is, indeed, the key factor for the sustainment of the enzymatic activity in saline environments. *Thioalkalivibrio* sp. *K90mix* is adapted to extreme salt concentrations and grows at salinities up to 4 M of NaCl. In a high salt concentration at least part of DNA should adapt Z form (Pohl and Jovin, 1972) and, if the C-terminal domain is adapted to bind this DNA form, then it is likely that the prokaryotic DNase is activated by the high ionic strength under the suboptimal concentrations of divalent ions. Thus, the dual peak of activity implies that the increasing salt concentration gradually suppresses the activity of this enzyme and the DNA binding domain is engaged only at higher salt concentrations thus rescuing the enzyme's ability to bind DNA.

Our experimental data (Table 2, Figure 3) implies that the C-terminal domain is indeed important for the adaptation of prokaryotic DNases to high ionic strength. We investigated this notion by creating two mutants of the prokaryotic DNase from *Thioalkalivibrio* sp. *K90mix* : the first mutant was generated by removing the C-terminal domain of the enzyme, the second mutant harbors an inactivating mutation in the active center of the DNase domain. The mutant with inactivating mutation in the active site of the DNase domain was created in order to test the hypothesis that the C-terminal domain acts only as a facilitator for the DNase domain, but cannot catalyze DNA hydrolysis by its own. The mutant with the removed C-terminal domain was created to test if this domain is required for the DNases activity at high ionic strength. The catalytic activity impairment of the active site mutant and the diminished salt tolerance of the mutant with the removed C-terminal domain was reliably confirmed by the data on digestion of short and long DNA substrates (Table 2, Figure 3). Therefore, DNA hydrolysation by the prokaryotic DNase is catalyzed by the active site in the DNase domain and the C-terminal domain acts as a facilitator at high salt concentrations (at least up to 4 M NaCl). Contrastingly, the bovine DNaseI is completely inhibited by NaCl concentrations above 1.2 M.

The modeled structures of DNases from two extremely halotolerant organisms *Thioalkalivibrio* sp. *K90mix* and *Methanohalobium evestigatum* as well as the subsequent electrostatic calculations indicated that the surfaces of these proteins are more electronegative compared to their eukaryotic counterparts (Figure 4). The adaptation of the enzyme to saline environments results in accumulation of negative amino acids on the surface (Siglioccolo et al., 2011; Graziano and Merlino, 2014). The data presented in Figure 4B clearly illustrates this trend: the extremely halophilic DNase from *Methanohalobium evestigatum* has the largest electronegative surface, the moderate halophilic DNase from *Thioalkalivibrio* sp. *K90mix* has a smaller area of the electronegative surface and the non-halotolerant bovine DNaseI has almost no electronegative areas on the surface of the protein side that is not facing DNA. Therefore, we have an indication that the prokaryotic DNaseI homologs tend to accumulate negatively charged surface residues during the adaptation to high salt concentrations and follow the tendency observed in other proteins (Siglioccolo et al., 2011; Graziano and Merlino, 2014).

A remarkable fact is that we observe accumulation of negatively charged residues in the DNA binding pocket of the protein (Figure 4A) when we compare DNaseTA (from extremely halotolerant organism) and bovine DNaseI. Analysis done by Becker et al. (2014) showed that such acidification of nucleic acid binding pocket was not observed in cases of TATA-binding protein and ribosome elongation factor, when proteins from halophilic and mesophilic organisms were compared. Also the DNA binding pocket did not show any sight of acidification comparing salt tolerant endonuclease I from *Vibrio salmonicida* and *Vibrio cholerae* (Niiranen et al., 2008). One would argue that this observed unusual acidification of DNaseTA's DNA binding pocket is due to adaptation for DNA binding at extremely high salt as it is in the case of PCNA from *Haloferax volcanii* (Morgunova et al., 2009). However, the PCNA is a complex trimeric DNA sliding protein and its DNA binding should greatly differ from enzymes or nucleic acid binding factors. Additionally, the DNase domain of DNaseTA without the aid of the C-terminal DNA binding domain is not salt tolerant (Figure 3, Table 2). Thus it is not likely, that the acidification of the DNaseTA DNA binding pocket is due to adaptation for DNA binding at high ionic strength. Therefore, it could be that the evolution was forced to find a compromise between a necessity to preserve DNA binding pocket from acidification, which would potentially disrupt DNA binding, and a necessity to adapt protein surface to high salt concentrations via additional negative charges. Nature's compromise was to enhance DNA binding via an additional DNA binding domain, which we experimentally proved to be responsible for the halotolerant properties of the DNase from *Thioalkalivibrio* sp. *K90mix*. As discussed above, such adaptation is characteristic to potentially secreted DNases produced by the micro-organisms living in hyper-saline environment.

Several attempts were made to enhance DNA binding properties of polymerases by fusing a HhH motifs containing domain (de Vega et al., 2010; Pavlov et al., 2012). Here we have shown that nature had similarly engineered DNases in response to confronting evolutionary pressures. These findings suggest that analogous engineering approaches could be explored to enhance the properties of eukaryotic DNases, which are widely used in biotechnology. However, this is another story to be told.

## Author contributions

GA performed phylogenetic analysis, formulated the hypothesis, did initial experiments and supervised further experiments and molecular modeling. MK performed the experimental work. AG did the molecular modeling tasks. AG and GA collected data from literature on salt tolerance of prokaryotes which is given in supplementary material. AL guided the whole work and design of experiments.

### Conflict of interest statement

This work was fully supported by Thermo Fisher Scientific Baltics UAB, V.A. Graiciuno 8, LT-02241 Vilnius, Lithuania. A related patent application is pending. The authors declare that the research was conducted in the absence of any commercial or financial relationships that could be construed as a potential conflict of interest.

## References

[B1] Altschul S. F. Madden T. L. Schäffer A. A. Zhang J. Zhang Z. Miller W.. (1997). Gapped blast and psi-blast: a new generation of protein database search programs. Nucleic Acids Res. 25, 3389–3402. 10.1093/nar/25.17.3389 9254694 PMC146917

[B2] Bagos P. G. Tsirigos K. D. Plessas S. K. Liakopoulos T. D. Hamodrakas S. J. (2009). Prediction of signal peptides in archaea. Protein Eng. Des. Sel. 22, 27–35. 10.1093/protein/gzn064 18988691

[B3] Baker N. A. Sept D. Joseph S. Holst M. J. McCammon J. A. (2001). Electrostatics of nanosystems: application to microtubules and the ribosome. Proc. Natl. Acad. Sci. U.S.A. 98, 10037–10041. 10.1073/pnas.181342398 11517324 PMC56910

[B4] Becker E. A. Seitzer P. M. Tritt A. Larsen D. Krusor M. Yao A. I.. (2014). Phylogenetically driven sequencing of extremely halophilic archaea reveals strategies for static and dynamic osmo-response. PLoS Genet. 10:e1004784. 10.1371/journal.pgen.1004784 25393412 PMC4230888

[B5] Benkert P. Tosatto S. C. Schomburg D. (2008). Qmean: A comprehensive scoring function for model quality assessment. Proteins Struct. Funct. Bioinformat. 71, 261–277. 10.1002/prot.21715 17932912

[B6] Bhardwaj G. Ko K. D. Hong Y. Zhang Z. Ho N. L. Chintapalli S. V.. (2012). PHYrn: a robust method for phylogenetic analysis of highly divergent sequences. PLoS ONE 7:e34261. 10.1371/journal.pone.0034261 22514627 PMC3325999

[B7] Bruns A. Rohde M. Berthe-Corti L. (2001). Muricauda ruestringensis gen. nov., sp. nov., a facultatively anaerobic, appendaged bacterium from german north sea intertidal sediment. Int. J. Syst. Evol. Microbiol. 51(Pt 6), 1997–2006. 10.1099/00207713-51-6-1997 11760940

[B8] Bruston F. Delbarre E. Ostlund C. Worman H. J. Buendia B. Duband-Goulet I. (2010). Loss of a dna binding site within the tail of prelamin a contributes to altered heterochromatin anchorage by progerin. FEBS Lett. 584, 2999–3004. 10.1016/j.febslet.2010.05.032 20580717 PMC2908524

[B9] Case D. Babin V. Berryman J. Betz R. Cai Q. Cerutti D. (2014). Amber 14.

[B10] Celniker G. Nimrod G. Ashkenazy H. Glaser F. Martz E. Mayrose I. (2013). Consurf: using evolutionary data to raise testable hypotheses about protein function. Israel J. Chem. 53, 199–206. 10.1002/ijch.201200096

[B11] Chen W.-J. Lai P.-J. Lai Y.-S. Huang P.-T. Lin C.-C. Liao T.-H. (2007). Probing the catalytic mechanism of bovine pancreatic deoxyribonuclease i by chemical rescue. Biochem. Biophys. Res. Commun. 352, 689–696. 10.1016/j.bbrc.2006.11.078 17141190

[B12] da Costa M. S. Santos H. Galinski E. A. (1998). An overview of the role and diversity of compatible solutes in bacteria and archaea. Adv. Biochem. Eng. Biotechnol. 61, 117–153. 10.1007/bfb0102291 9670799

[B13] de Vega M. Lázaro J. M. Mencía M. Blanco L. Salas M. (2010). Improvement of ϕ29 dna polymerase amplification performance by fusion of dna binding motifs. Proc. Natl. Acad. Sci. U.S.A. 107, 16506–16511. 10.1073/pnas.1011428107 20823261 PMC2944734

[B14] Doherty A. J. Serpell L. C. Ponting C. P. (1996). The helix-hairpin-helix dna-binding motif: a structural basis for non-sequence-specific recognition of dna. Nucleic Acids Res. 24, 2488–2497. 10.1093/nar/24.13.2488 8692686 PMC145986

[B15] Dolinsky T. J. Nielsen J. E. McCammon J. A. Baker N. A. (2004). Pdb2pqr: an automated pipeline for the setup of poisson–boltzmann electrostatics calculations. Nucleic Acids Res. 32(Suppl. 2), W665–W667. 10.1093/nar/gkh381 15215472 PMC441519

[B16] Eddy S. R. (2011). Accelerated profile hmm searches. PLoS Comput. Biol. 7:e1002195. 10.1371/journal.pcbi.1002195 22039361 PMC3197634

[B17] Felsenstein J. (1989). Phylip - phylogeny inference package (version 3.2). Cladistics 5, 164–166.

[B18] Garcia-Diaz M. Bebenek K. Krahn J. M. Blanco L. Kunkel T. A. Pedersen L. C. (2004). A structural solution for the dna polymerase lambda-dependent repair of dna gaps with minimal homology. Mol. Cell 13, 561–572. 10.1016/S1097-2765(04)00061-9 14992725

[B19] Graziano G. Merlino A. (2014). Molecular bases of protein halotolerance. Biochim. Biophys. Acta 1844, 850–858. 10.1016/j.bbapap.2014.02.018 24590113

[B20] Guéroult M. Picot D. Abi-Ghanem J. Hartmann B. Baaden M. (2010). How cations can assist dnase i in dna binding and hydrolysis. PLoS Comput. Biol. 6:e1001000. 10.1371/journal.pcbi.1001000 21124947 PMC2987838

[B21] Holm L. Rosenström P. (2010). Dali server: conservation mapping in 3d. Nucleic Acids Res. 38(Suppl. 2), W545–W549. 10.1093/nar/gkq366 20457744 PMC2896194

[B22] Huerta-Cepas J. Dopazo J. Gabaldón T. (2010). Ete: a python environment for tree exploration. BMC Bioinformatics 11:24. 10.1186/1471-2105-11-24 20070885 PMC2820433

[B23] Hunter S. Jones P. Mitchell A. Apweiler R. Attwood T. K. Bateman A.. (2012). Interpro in 2011: new developments in the family and domain prediction database. Nucleic Acids Res. 40, D306–D312. 10.1093/nar/gks456 22096229 PMC3245097

[B24] Jones P. Binns D. Chang H.-Y. Fraser M. Li W. McAnulla C.. (2014). Interproscan 5: genome-scale protein function classification. Bioinformatics 30, 1236–1240. 10.1093/bioinformatics/btu031 24451626 PMC3998142

[B25] Kamekura M. Onishi H. (1974). Halophilic nuclease from a moderately halophilic micrococcus varians. J. Bacteriol. 119, 339–344. 4852218 10.1128/jb.119.2.339-344.1974 PMC245612

[B26] Kamekura M. Onishi H. (1978). Properties of the halophilic nuclease of a moderate halophile, micrococcus varians subsp. halophilus. J. Bacteriol. 133, 59–65. 618848 10.1128/jb.133.1.59-65.1978 PMC221976

[B27] Kanlayakrit W. Ikeda T. Tojai S. Rodprapakorn M. Sirisansaneeyakul S. (2001). Isolation and characterization of extracellular halophilic ribonuclease from halotolerant pseudomonas species. Kasetsart J. 35, 179–187.

[B28] Katoh K. Standley D. M. (2013). Mafft multiple sequence alignment software version 7: improvements in performance and usability. Mol. Biol. Evol. 30, 772–780. 10.1093/molbev/mst010 23329690 PMC3603318

[B29] Katoh K. Toh H. (2008). Recent developments in the mafft multiple sequence alignment program. Briefings Bioinf. 9, 286–298. 10.1093/bib/bbn013 18372315

[B30] Koechler S. Plewniak F. Barbe V. Battaglia-Brunet F. Jost B. Joulian C.. (2013). Genome sequence of halomonas sp. strain a3h3, isolated from arsenic-rich marine sediments. Genome Announ. 1:e00819–13. 10.1128/genomeA.00819-13 24115546 PMC3795216

[B31] Koops H.-P. ttcher B. Mller U. Pommerening-Rser A. Stehr G. (1990). Description of a new species of nitrosococcus. Arch. Microbiol. 154, 244–248. 10.1007/BF00248962

[B32] Lin Y. Fan H. Hao X. Johnstone L. Hu Y. Wei G.. (2012). Draft genome sequence of halomonas sp. strain hal1, a moderately halophilic arsenite-oxidizing bacterium isolated from gold-mine soil. J. Bacteriol. 194, 199–200. 10.1128/JB.06359-11 22156396 PMC3256601

[B33] Mans B. J. Anantharaman V. Aravind L. Koonin E. V. (2004). Comparative genomics, evolution and origins of the nuclear envelope and nuclear pore complex. Cell Cycle 3, 1612–1637. 10.4161/cc.3.12.1316 15611647

[B34] Martínez-Cánovas M. J. Quesada E. Martínez-Checa F. del Moral A. Béjar V. (2004). Salipiger mucescens gen. nov., sp. nov., a moderately halophilic, exopolysaccharide-producing bacterium isolated from hypersaline soil, belonging to the α-proteobacteria. Int. J. Syst. Evol. Microbiol. 54, 1735–1740. 10.1099/ijs.0.63166-0 15388737

[B35] Morgunova E. Gray F. C. Macneill S. A. Ladenstein R. (2009). Structural insights into the adaptation of proliferating cell nuclear antigen (pcna) from haloferax volcanii to a high-salt environment. Acta Crystallogr. D Biol. Crystallogr. 65(Pt 10), 1081–1088. 10.1107/S0907444909029321 19770505 PMC2756170

[B36] Muyzer G. Sorokin D. Y. Mavromatis K. Lapidus A. Foster B. Sun H. (2011). Complete genome sequence of thioalkalivibrio sp. k90mix. Stan. Genom. Sci. 5:341 10.4056/sigs.2315092 PMC3368412 22675584

[B37] Niiranen L. Altermark B. Brandsdal B. O. Leiros H.-K. S. Helland R. Smalås A. O.. (2008). Effects of salt on the kinetics and thermodynamic stability of endonuclease i from vibrio salmonicida and vibrio cholerae. FEBS J. 275, 1593–1605. 10.1111/j.1742-4658.2008.06317.x 18312415

[B38] Onishi H. Mori T. Takeuchi S. Tani K. Kobayashi T. Kamekura M. (1983). Halophilic nuclease of a moderately halophilic bacillus sp.: Production, purification, and characterization. Appl. Environ. Microbiol. 45, 24–30. 16346168 10.1128/aem.45.1.24-30.1983 PMC242226

[B39] Pan C. Q. Lazarus R. A. (1998). Hyperactivity of human dnase i variants. dependence on the number of positively charged residues and concentration, length, and environment of dna. J. Biol. Chem. 273, 11701–11708. 10.1074/jbc.273.19.11701 9565591

[B40] Pavlov A. R. Pavlova N. V. Kozyavkin S. A. Slesarev A. I. (2012). Cooperation between catalytic and dna binding domains enhances thermostability and supports dna synthesis at higher temperatures by thermostable dna polymerases. Biochemistry 51, 2032–2043. 10.1021/bi2014807 22320201 PMC3345285

[B41] Petersen T. N. Brunak S. von Heijne G. Nielsen H. (2011). Signalp 4.0: discriminating signal peptides from transmembrane regions. Nat. Methods 8, 785–786. 10.1038/nmeth.1701 21959131

[B42] Pohl F. M. Jovin T. M. (1972). Salt-induced co-operative conformational change of a synthetic dna: equilibrium and kinetic studies with poly (dg-dc). J. Mol. Biol. 67, 375–396. 10.1016/0022-2836(72)90457-3 5045303

[B43] Remmert M. Biegert A. Hauser A. Söding J. (2012). Hhblits: lightning-fast iterative protein sequence searching by hmm-hmm alignment. Nat. Methods 9, 173–175. 10.1038/nmeth.1818 22198341

[B44] Reynolds S. M. Käll L. Riffle M. E. Bilmes J. A. Noble W. S. (2008). Transmembrane topology and signal peptide prediction using dynamic bayesian networks. PLoS Comput. Biol. 4:e1000213. 10.1371/journal.pcbi.1000213 18989393 PMC2570248

[B45] Rodrigues J. P. Levitt M. Chopra G. (2012). Kobamin: a knowledge-based minimization web server for protein structure refinement. Nucleic Acids Res. 40, W323–W328. 10.1093/nar/gks376 22564897 PMC3394243

[B46] Roy A. Kucukural A. Zhang Y. (2010). I-tasser: a unified platform for automated protein structure and function prediction. Nat. Protocols 5, 725–738. 10.1038/nprot.2010.5 PMC2849174 20360767

[B47] Schlesner H. (1989). Planctomyces brasiliensis sp. nov., a halotolerant bacterium from a salt pit. Syst. Appl. Microbiol. 12, 159–161. 10.1016/S0723-2020(89)80008-6

[B48] Siglioccolo A. Paiardini A. Piscitelli M. Pascarella S. (2011). Structural adaptation of extreme halophilic proteins through decrease of conserved hydrophobic contact surface. BMC Struct. Biol. 11:50. 10.1186/1472-6807-11-50 22192175 PMC3293032

[B49] Singh S. Folkers G. E. Bonvin A. M. J. J. Boelens R. Wechselberger R. Niztayev A.. (2002). Solution structure and dna-binding properties of the c-terminal domain of uvrc from *E. coli*. EMBO J. 21, 6257–6266. 10.1093/emboj/cdf627 12426397 PMC137216

[B50] Tan D. Xue Y.-S. Aibaidula G. Chen G.-Q. (2011). Unsterile and continuous production of polyhydroxybutyrate by halomonas td01. Biores. Technol. 102, 8130–8136. 10.1016/j.biortech.2011.05.068 21680179

[B51] UniProt Consortium. (2014). Activities at the universal protein resource (uniprot). Nucleic Acids Res. 42, D191–D198. 10.1093/nar/gkt1140 24253303 PMC3965022

[B52] Wiederstein M. Sippl M. J. (2007). Prosa-web: interactive web service for the recognition of errors in three-dimensional structures of proteins. Nucleic Acids Res. 35(Suppl. 2), W407–W410. 10.1093/nar/gkm290 17517781 PMC1933241

[B53] Wilharm T. Zhilina T. Hummel P. (1991). Dna-dna hybridization of methylotrophic halophilic methanogenic bacteria and transfer of methanococcus halophilusvp to the genus methanohalophilus as methanohalophilus halophilus comb. nov. Int. J. Syst. Bacteriol. 41, 558–562.

