# Supplementary material for: Domain organization of DNase from Thioalkalivibrio sp. provides insights into retention of activity in high salt environments
Source: Front Microbiol. 2015 Jul 1;6:661. doi: 10.3389/fmicb.2015.00661 (PMC4486849; doi:10.3389/fmicb.2015.00661)
Supplement: Supplementary file 1 [file Table1.PDF]

Supplementary Material: Domain organization of DNase from *Thioalkalivibrio sp.* suggests insights on how bacterial DNases can retain activity at extremes of ionic strength

Gediminas Alzbutas [1,2,*], Milda Kaniusaite [2], Algirdas Grybauskas [2,3] and Arunas Lagunavicius [2]

[1] *VU Institute of Biotechnology, V.A. Graiciuno 8, LT-02241 Vilnius, Lithuania*
[2] *Thermo Fisher Scientific, V.A. Graiciuno 8, LT-02241 Vilnius, Lithuania*
[3] *Vilnius University, Universiteto str. 3 LT-01513 Vilnius, Lithuania*

Correspondence*:
Gediminas Alzbutas
Thermo Fisher Scientific, V.A. Graiciuno 8, LT-02241 Vilnius, Lithuania,
gediminas.alzbutas@thermofisher.com

Extremophilic Industrially Important Enzymes and Molecular Mechanisms

1 SUPPLEMENTARY TABLES AND FIGURES

Supplementary Table S1. Sequences of oligonucleotides which were used to construct mutants of DNaseTA.

DNaseTA mutant	Primer	PCR step	Sequence
DNaseTA H134A	Forward	1	GCCGCCACGGTTGCAATCACCTACGG
	Reverse		GTGGTGGTGATGGTGATGGCCCGGCTCGATGCAGGCCTCAC
	Reverse	2	AGAAGGAGATATAACTATGCTGCGCCTGGCAAGCTGG
DNaseTA Δ C mutant	Forward	1	GTGGTGGTGATGGTGATGGCCACCTTCGAACAGTACATAAACCGG
	Reverse		AGAAGGAGATATAACTATGCTGCGCCTGGCAAGC
