# Supplementary material for: Domain organization of DNase from Thioalkalivibrio sp. provides insights into retention of activity in high salt environments
Source: Front Microbiol. 2015 Jul 1;6:661. doi: 10.3389/fmicb.2015.00661 (PMC4486849; doi:10.3389/fmicb.2015.00661)
Supplement: Supplementary file 2 [file Table2.PDF]

Supplementary Material: Domain organization of DNase from *Thioalkalivibrio sp.* provides insights into retention of activity in high salt environments

Gediminas Alzbutas [1,2,*], Milda Kaniusaite [2], Algirdas Grybauskas [2,3] and Arunas Lagunavicius [2]

[1] *VU Institute of Biotechnology, V.A. Graiciuno 8, LT-02241 Vilnius, Lithuania*
[2] *Thermo Fisher Scientific, V.A. Graiciuno 8, LT-02241 Vilnius, Lithuania*
[3] *Vilnius University, Universiteto str. 3 LT-01513 Vilnius, Lithuania*

Correspondence*:
Gediminas Alzbutas
Thermo Fisher Scientific, V.A. Graiciuno 8, LT-02241 Vilnius, Lithuania,
gediminas.alzbutas@thermofisher.com

Extremophilic Industrially Important Enzymes and Molecular Mechanisms

1 SUPPLEMENTARY TABLES AND FIGURES

Supplementary Table S2. Collected data on salt tolerance of procariotic organisms, which have DNaseI family proteins

Representative species/label used in phylogeny tree	UniRef90 cluster (an accession number of a representative sequence)	Max. NaCl conc., M	Halotolerance level	Publications used for data inference
Bizionia argentinensis JUB59	G2EGF9	1.00	medium	**Bercovich et al.** (2008)
Blastopirellula marina DSM 3645	A3ZMZ4, A3ZS40	1.00	medium	**Schlesner et al.** (2004)
Cetobacterium somerae ATCC BAA-474	U7VDD6	0.00	low	**Finegold et al.** (2003)
Corallococcus coralloides ATCC 25202	H8MMN3	0.17	low	**Stackebrandt et al.** (2007)
Deinococcus deserti VCD115	C1D1Q7	0.10	low	**De Groot et al.** (2005)
Deinococcus geothermalis DSM 11300	Q1IZR2	0.00	low	**Ferreira et al.** (1997)
Deinococcus maricopensis DSM 21211	E8U979	0.00	low	**Pukall et al.** (2011)
Deinococcus proteolyticus ATCC 35074	F0RL87	0.85	medium	**Copeland et al.** (2012)
Deinococcus radiodurans ATCC 13939	Q9RZQ3	0.00	low	**Thompson and Murray** (1981)
Fibrisoma limi BUZ 3	I2GRN0	0.34	slight	**Filippini et al.** (2011)
Flavobacterium branchiophilum FL-15	G2Z3P7	0.00	low	**Touchon et al.** (2011)
Flavobacterium frigoris PS1	H7FLG2	0.85	medium	**Van Trappen et al.** (2004a)
Flavobacterium indicum DSM 17447	H8XPU7	0.34	slight	**Saha and Chakrabarti** (2006)
Flavobacterium johnsoniae ATCC 17061	A5FNZ6	0.00	low	**McBride and Kempf** (1996)
Flavobacterium sp. CF136	J3BY94	0.00	low	**Kolton et al.** (2013)
Flavobacterium sp. F52	J0RRS2	0.00	low	**Kolton et al.** (2013)
Flexibacter litoralis ATCC 23117	I4ALP5	0.00	slight	**Suzuki et al.** (2001)
Formosa sp. AK20	M7MKE5	0.00	slight	**Mann et al.** (2013)
Fusobacterium mortiferum ATCC 9817	C3WAK9	0.00	low	**Bjornson and Hill** (1973)
Fusobacterium necrophorum 1_1_36S	H1D400	0.00	low	**Bumgarner and Finkelstein** (1973)
Fusobacterium necrophorum ATCC 51357	I3DDW2	0.00	low	**Bumgarner and Finkelstein** (1973)
Fusobacterium necrophorum D12	E5BLB3	0.00	low	**Bumgarner and Finkelstein** (1973)
Fusobacterium nucleatum 4_8	R9RDT2	0.00	low	**Triches et al.** (2014)
Fusobacterium nucleatum ATCC 25586	Q8R5P1	0.00	low	**Triches et al.** (2014)
Fusobacterium periodonticum 2_1_31	C3WGZ0	0.00	low	**Triches et al.** (2014)
Fusobacterium sp. F0437	G6C0F0	0.00	low	**Huggan and Murdoch** (2008)
Fusobacterium varium ATCC 27725	C6JIF4	0.00	low	**Huggan and Murdoch** (2008)
Gillisia limnaea DSM 15749	H2BXJ8	0.85	medium	**Van Trappen et al.** (2004b); **Riedel et al.** (2012)
Halomonas sp. A3H3	T2LFY5	1.50	medium	**Koechler et al.** (2013)
Halomonas sp. HAL1	G4F511	2.00	medium	**Lin et al.** (2012)
Halomonas sp. PBN3	U7P800	0.00	slight	**Overholt et al.** (2013)

Continued on next page

Halomonas sp. TD01	F7SPZ3	3.42	extreme	**Cai et al.** (2011); **Tan et al.** (2011)
Ilyobacter polytropus DSM 2926	E3H9Z7	0.00	low	**Sikorski et al.** (2010)
Kordia algicida OT-1	A9DNF0	0.85	medium	**Sohn et al.** (2004)
Leptospira interrogans str, FPW2026	J4U625	0.00	low	**Ren et al.** (2003)
Leptotrichia buccalis ATCC 14201	C7N942	0.00	low	**Hamilton and Zahler** (1957)
Leptotrichia goodfellowii F0264	D0GJB4	0.00	low	**Eribe et al.** (2004)
Leptotrichia hofstadii F0254	C9MV32	0.00	low	**Eribe et al.** (2004)
Leptotrichia sp. F0557	U2Q3R8	0.00	low	**Huggan and Murdoch** (2008)
Leptotrichia sp. W9775	U2SK95	0.00	low	**Eribe et al.** (2004)
Leptotrichia wadei F0279	U2PRB5	0.00	low	**Eribe et al.** (2004)
Meiothermus silvanus ATCC 700542	D7BD91	0.20	low	**Sikorski et al.** (2010)
Methanococcoides burtonii DSM 6242	Q12YY7	0.50	slight	**Franzmann et al.** (1992)
Methanohalobium evestigatum DSM 3721	D7E828	5.10	extreme	**Wilharm et al.** (1991)
Methanoplanus limicola DSM 2279	H1Z285	0.92	medium	**Wildgruber et al.** (1982)
Methanoplanus petrolearius DSM 11571	E1RK68	0.85	medium	**Ollivier et al.** (1997)
Methanosarcina barkeri Fusaro	Q467S8	0.00	low	**Maeder et al.** (2006)
Myroides odoratimimus CCUG 3837	K1HGB8	1.03	medium	**Yoon et al.** (2006a)
Myroides odoratus DSM 2801	H1Z8R7	0.86	medium	**Yoon et al.** (2006a)
Myxococcus sp.	U2SKR7	0.26	low	**Lang and Stackebrandt** (2009)
Muricauda ruestringensis DSM 13258	G2PLU4, G2PQ99	1.54	medium	**Bruns et al.** (2001)
Nitrosococcus halophilus Nc4	D5C5F3	1.60	medium	**Campbell et al.** (2011); **Koops et al.** (1990)
Nitrosococcus watsoni C-113	D8KCF8	0.80	medium	**Campbell et al.** (2011)
Nonlabens dokdonensis DSM 17205	L7WAP7	1.37	medium	**Kwon et al.** (2013); **Yoon et al.** (2006b)
Pedobacter sp. BAL39	A6EH02	0.50	slight	**Han et al.** (2009)
Pirellula staleyi ATCC 27377	D2R4L4	0.30	low	**Schlesner et al.** (2004)
Planctomyces brasiliensis ATCC 49424	F0SFA3	1.70	medium	**Schlesner** (1989)
Planctomyces maris DSM 8797	A6C718	0.70	slight	**Schlesner** (1989)
Pseudomonas aeruginosa 39016	E2ZVF4	0.00	low	**Itah and Essien** (2005)
Pseudomonas putida S12	V4H6S2	0.50	slight	**Kets et al.** (1996)
Pseudomonas syringae ES4326	F3HK83	0.00	low	**Hendrickson et al.** (2000)
Pseudomonas sp. M47T1	I4MVP4	0.00	low	**Proença et al.** (2012)
Psychroflexus torquis ATCC 700755	K4IGL3	0.80	medium	**Bowman et al.** (1998)
Rhodopirellula baltica SH1	Q7UV93	1.20	medium	**Schlesner et al.** (2004)
Rhodopirellula baltica WH47	F2AS22	1.20	medium	**Schlesner et al.** (2004)
Rhodopirellula maiorica SM1	M5RC55, M5S411	0.00	slight	**Wegner et al.** (2014)
Rhodopirellula sallentina SM41	M5UED4	0.00	slight	**Wegner et al.** (2014)
Rhodopirellula sp. SWK7	M5T112, M5TEL8	0.00	slight	**Wegner et al.** (2014)

Continued on next page

Salipiger mucosus DSM 16094	S9QWK2	3.42	extreme	**Martínez-Cánovas et al.** (2004)
Sebaldella termitidis ATCC 33386	D1AKL2	0.00	low	**Harmon-Smith et al.** (2010)
Simiduia agarivorans DSM 21679	K4KHZ4	1.19	medium	**Shieh et al.** (2008)
Streptobacillus moniliformis ATCC 14647	D1AYT4	0.00	low	**Nolan et al.** (2009)
Thaumarchaeota archaeon SCGC AB-539-E09	M7TTD1	0.00	slight	**Muller et al.** (2010)
Thermus sp. CCB_US3_UF1	G8N853	0.00	slight	**Teh et al.** (2012)
Thermus thermophilus HB8	G9MB93	0.86	medium	**Nunes et al.** (1995)
Thermus thermophilus JL-18	H9ZV47	0.00	low	**Murugapiran et al.** (2013)
Thioalkalivibrio sp. K90mix	D3SGB1	4.00	extreme	**Muyzer et al.** (2011)
Treponema azotonutricium ATCC BAA-888	F5YE44	0.00	low	**Graber and Breznak** (2004); **Vu et al.** (2004)
Treponema primitia ATCC BAA-887	F5YJE1	0.00	low	**Graber et al.** (2004); **Vu et al.** (2004)
unidentified eubacterium SCB49	A6EMT3	0.00	slight	**Moore Foundation** (2010)
Vibrio coralliilyticus ATCC BAA-450	C9NPE5	1.20	medium	**Ben-Haim et al.** (2003a,b)
Zobellia galactanivorans DSM 12802	G0LBP4	0.00	slight	**Thomas et al.** (2011)

REFERENCES

Ben-Haim, Y., Thompson, F., Thompson, C., Cnockaert, M., Hoste, B., Swings, J., et al. (2003a), Vibrio coralliilyticus sp. nov., a temperature-dependent pathogen of the coral pocillopora damicornis, *International Journal of Systematic and Evolutionary Microbiology*, 53, 1, 309–315

Ben-Haim, Y., Thompson, F. L., Thompson, C. C., Cnockaert, M. C., Hoste, B., Swings, J., et al. (2003b), Vibrio coralliilyticus sp. nov., a temperature-dependent pathogen of the coral pocillopora damicornis., *Int J Syst Evol Microbiol*, 53, Pt 1, 309–315

Bercovich, A., Vazquez, S. C., Yankilevich, P., Coria, S. H., Foti, M., Hernández, E., et al. (2008), Bizionia argentinensis sp. nov., isolated from surface marine water in antarctica, *International journal of systematic and evolutionary microbiology*, 58, 10, 2363–2367

Bjornson, H. and Hill, E. (1973), Bacteroidaceae in thromboembolic disease: effects of cell wall components on blood coagulation in vivo and in vitro, *Infection and immunity*, 8, 6, 911–918

Bowman, J. P., McCammon, S. A., Lewis, T., Skerratt, J. H., Brown, J. L., Nichols, D. S., et al. (1998), Psychroflexus torquis gen. nov., sp. nov. a psychrophilic species from antarctic sea ice, and reclassification of flavobacterium gondwanense (dobson et al. 1993) as psychroflexus gondwanense gen. nov., comb. nov., *Microbiology*, 144, 6, 1601–1609

Bruns, A., Rohde, M., and Berthe-Corti, L. (2001), Muricauda ruestringensis gen. nov., sp. nov., a facultatively anaerobic, appendaged bacterium from german north sea intertidal sediment., *Int J Syst Evol Microbiol*, 51, Pt 6, 1997–2006

Bumgarner, L. R. and Finkelstein, R. A. (1973), Pathogenesis and immunology of experimental gonococcal infection: virulence of colony types of neisseria gonorrhoeae for chicken embryos, *Infection and immunity*, 8, 6, 919–924

Cai, L., Tan, D., Aibaidula, G., Dong, X.-R., Chen, J.-C., Tian, W.-D., et al. (2011), Comparative genomics study of polyhydroxyalkanoates (pha) and ectoine relevant genes from halomonas sp. td01 revealed extensive horizontal gene transfer events and co-evolutionary relationships, *Microb Cell Fact*, 10, 88

Campbell, M. A., Chain, P. S., Dang, H., El Sheikh, A. F., Norton, J. M., Ward, N. L., et al. (2011), Nitrosococcus watsonii sp. nov., a new species of marine obligate ammonia-oxidizing bacteria that is not omnipresent in the world's oceans: calls to validate the names nitrosococcus halophilus and nitrosomonas mobilis, *FEMS microbiology ecology*, 76, 1, 39–48

Copeland, A., Zeytun, A., Yassawong, M., Nolan, M., Lucas, S., Hammon, N., et al. (2012), Complete genome sequence of the orange-red pigmented, radioresistant deinococcus proteolyticus type strain (mrpt), *Standards in genomic sciences*, 6, 2, 240

De Groot, A., Chapon, V., Servant, P., Christen, R., Fischer-Le Saux, M., Sommer, S., et al. (2005), Deinococcus deserti sp. nov., a gamma-radiation-tolerant bacterium isolated from the sahara desert, *International journal of systematic and evolutionary microbiology*, 55, 6, 2441–2446

Eribe, E. R., Paster, B. J., Caugant, D. A., Dewhirst, F. E., Stromberg, V. K., Lacy, G. H., et al. (2004), Genetic diversity of leptotrichia and description of leptotrichia goodfellowii sp. nov., leptotrichia hofstadii sp. nov., leptotrichia shahii sp. nov. and leptotrichia wadei sp. nov., *International journal of systematic and evolutionary microbiology*, 54, 2, 583–592

Ferreira, A. C., Nobre, M. F., Rainey, F. A., Silva, M. T., Wait, R., Burghardt, J., et al. (1997), Deinococcus geothermalis sp. nov. and deinococcus murrayi sp. nov., two extremely radiation-resistant and slightly thermophilic species from hot springs, *International journal of systematic bacteriology*, 47, 4, 939–947

Filippini, M., Kaech, A., Ziegler, U., and Bagheri, H. C. (2011), Fibrisoma limi gen. nov., sp. nov., a filamentous bacterium isolated from tidal flats, *International journal of systematic and evolutionary microbiology*, 61, 6, 1418–1424

Finegold, S. M., Vaisanen, M.-L., Molitoris, D. R., Tomzynski, T. J., Song, Y., Liu, C., et al. (2003), Cetobacterium somerae sp. nov. from human feces and emended description of the genus cetobacterium., *Syst Appl Microbiol*, 26, 2, 177–181, doi:10.1078/072320203322346010

Franzmann, P., Springer, N., Ludwig, W., Conway de Macario, E., and Rohde, M. (1992), A methanogenic archaeon from ace lake, antarctica: Methanococcoides burtonii sp. nov., *Systematic and applied microbiology*, 15, 4, 573–581

Graber, J. R. and Breznak, J. A. (2004), Physiology and nutrition of treponema primitia, an h2/co2-acetogenic spirochete from termite hindguts, *Applied and environmental microbiology*, 70, 3, 1307–1314

Graber, J. R., Leadbetter, J. R., and Breznak, J. A. (2004), Description of treponema azotonutricium sp. nov. and treponema primitia sp. nov., the first spirochetes isolated from termite guts., *Appl Environ Microbiol*, 70, 3, 1315–1320

Hamilton, R. D. and Zahler, S. A. (1957), A study of leptotrichia buccalis, *Journal of bacteriology*, 73, 3, 386

Han, C., Spring, S., Lapidus, A., Del Rio, T. G., Tice, H., Copeland, A., et al. (2009), Complete genome sequence of pedobacter heparinus type strain (him 762-3t), *Standards in genomic sciences*, 1, 1, 54

Harmon-Smith, M., Celia, L., Chertkov, O., Lapidus, A., Copeland, A., Del Rio, T. G., et al. (2010), Complete genome sequence of sebaldella termitidis type strain (nctc 11300t), *Standards in genomic sciences*, 2, 2, 220

Hendrickson, E. L., Guevera, P., Peñaloza-Vàzquez, A., Shao, J., Bender, C., and Ausubel, F. M. (2000), Virulence of the phytopathogen pseudomonas syringae pv. maculicola is rpon dependent, *Journal of bacteriology*, 182, 12, 3498–3507

Huggan, P. J. and Murdoch, D. R. (2008), Fusobacterial infections: clinical spectrum and incidence of invasive disease., *J Infect*, 57, 4, 283–289, doi:10.1016/j.jinf.2008.07.016

Itah, A. and Essien, J. (2005), Growth profile and hydrocarbonoclastic potential of microorganisms isolated from tarballs in the bight of bonny, nigeria, *World Journal of Microbiology and Biotechnology*, 21, 6-7, 1317–1322

Kets, E. P., Galinski, E. A., de Wit, M., de Bont, J. A., and Heipieper, H. J. (1996), Mannitol, a novel bacterial compatible solute in pseudomonas putida s12., *J Bacteriol*, 178, 23, 6665–6670

Koechler, S., Plewniak, F., Barbe, V., Battaglia-Brunet, F., Jost, B., Joulian, C., et al. (2013), Genome sequence of halomonas sp. strain a3h3, isolated from arsenic-rich marine sediments, *Genome announcements*, 1, 5, e00819–13

Kolton, M., Sela, N., Elad, Y., and Cytryn, E. (2013), Comparative genomic analysis indicates that niche adaptation of terrestrial flavobacteria is strongly linked to plant glycan metabolism, *PloS one*, 8, 9, e76704

Koops, H.-P., Bttcher, B., Mller, U., Pommerening-Rser, A., and Stehr, G. (1990), Description of a new species of nitrosococcus, *Archives of Microbiology*, 154, 3, 244–248, doi:10.1007/BF00248962

Kwon, S.-K., Kim, B. K., Song, J. Y., Kwak, M.-J., Lee, C. H., Yoon, J.-H., et al. (2013), Genomic makeup of the marine flavobacterium nonlabens (donghaeana) dokdonensis and identification of a novel class of rhodopsins, *Genome biology and evolution*, 5, 1, 187–199

Lang, E. and Stackebrandt, E. (2009), Emended descriptions of the genera myxococcus and corallococcus, typification of the species myxococcus stipitatus and myxococcus macrosporus and a proposal that they be represented by neotype strains. request for an opinion, *International journal of systematic and evolutionary microbiology*, 59, 8, 2122–2128

Lin, Y., Fan, H., Hao, X., Johnstone, L., Hu, Y., Wei, G., et al. (2012), Draft genome sequence of halomonas sp. strain hal1, a moderately halophilic arsenite-oxidizing bacterium isolated from gold-mine soil, *Journal of bacteriology*, 194, 1, 199–200

Maeder, D. L., Anderson, I., Brettin, T. S., Bruce, D. C., Gilna, P., Han, C. S., et al. (2006), The methanosarcina barkeri genome: comparative analysis with methanosarcina acetivorans and methanosarcina mazei reveals extensive rearrangement within methanosarcinal genomes, *Journal of bacteriology*, 188, 22, 7922–7931

Mann, A. J., Hahnke, R. L., Huang, S., Werner, J., Xing, P., Barbeyron, T., et al. (2013), The genome of the alga-associated marine flavobacterium formosa agariphila kmm 3901t reveals a broad potential for degradation of algal polysaccharides, *Applied and environmental microbiology*, 79, 21, 6813–6822

Martínez-Cánovas, M. J., Quesada, E., Martínez-Checa, F., del Moral, A., and Béjar, V. (2004), Salipiger mucescens gen. nov., sp. nov., a moderately halophilic, exopolysaccharide-producing bacterium isolated from hypersaline soil, belonging to the α-proteobacteria, *International journal of systematic and evolutionary microbiology*, 54, 5, 1735–1740

McBride, M. and Kempf, M. (1996), Development of techniques for the genetic manipulation of the gliding bacterium cytophaga johnsonae., *Journal of bacteriology*, 178, 3, 583–590

Moore Foundation, J. C. V. I. (2010), Bioproject entry accession: Prjna54737

Muller, F., Brissac, T., Le Bris, N., Felbeck, H., and Gros, O. (2010), First description of giant archaea (thaumarchaeota) associated with putative bacterial ectosymbionts in a sulfidic marine habitat, *Environmental microbiology*, 12, 8, 2371–2383

Murugapiran, S. K., Huntemann, M., Wei, C.-L., Han, J., Detter, J. C., Han, C., et al. (2013), Thermus oshimai jl-2 and t. thermophilus jl-18 genome analysis illuminates pathways for carbon, nitrogen, and sulfur cycling, *Standards in genomic sciences*, 7, 3, 449

Muyzer, G., Sorokin, D. Y., Mavromatis, K., Lapidus, A., Foster, B., Sun, H., et al. (2011), Complete genome sequence of thioalkalivibrio sp. k90mix, *Standards in genomic sciences*, 5, 3, 341

Nolan, M., Gronow, S., Lapidus, A., Ivanova, N., Copeland, A., Lucas, S., et al. (2009), Complete genome sequence of streptobacillus moniliformis type strain (9901t), *Standards in genomic sciences*, 1, 3, 300

Nunes, O. C., Manaia, C. M., Da Costa, M. S., and Santos, H. (1995), Compatible solutes in the thermophilic bacteria rhodothermus marinus and "thermus thermophilus"., *Appl Environ Microbiol*, 61, 6, 2351–2357

Ollivier, B., Cayol, J.-L., Patel, B., Magot, M., Fardeau, M.-L., and Garcia, J.-L. (1997), Methanoplanus petrolearius sp. nov., a novel methanogenic bacterium from an oil-producing well, *FEMS microbiology letters*, 147, 1, 51–56

Overholt, W. A., Green, S. J., Marks, K. P., Venkatraman, R., Prakash, O., and Kostka, J. E. (2013), Draft genome sequences for oil-degrading bacterial strains from beach sands impacted by the deepwater horizon oil spill, *Genome announcements*, 1, 6, e01015–13

Proença, D. N., Espírito Santo, C., Grass, G., and Morais, P. V. (2012), Draft genome sequence of pseudomonas sp. strain m47t1, carried by bursaphelenchus xylophilus isolated from pinus pinaster., *J Bacteriol*, 194, 17, 4789–4790, doi:10.1128/JB.01116-12

Pukall, R., Zeytun, A., Lucas, S., Lapidus, A., Hammon, N., Deshpande, S., et al. (2011), Complete genome sequence of deinococcus maricopensis type strain (lb-34t), *Standards in genomic sciences*, 4, 2, 163

Ren, S.-X., Fu, G., Jiang, X.-G., Zeng, R., Miao, Y.-G., Xu, H., et al. (2003), Unique physiological and pathogenic features of leptospira interrogans revealed by whole-genome sequencing, *Nature*, 422, 6934, 888–893

Riedel, T., Held, B., Nolan, M., Lucas, S., Lapidus, A., Tice, H., et al. (2012), Genome sequence of the antarctic rhodopsins-containing flavobacterium gillisia limnaea type strain (r-8282(t))., *Stand Genomic Sci*, 7, 1, 107–119, doi:10.4056/sigs.3216895

Saha, P. and Chakrabarti, T. (2006), Flavobacterium indicum sp. nov., isolated from warm spring water in assam, india, *International journal of systematic and evolutionary microbiology*, 56, 11, 2617–2621

Schlesner, H. (1989), Planctomyces brasiliensis sp. nov., a halotolerant bacterium from a salt pit, *Systematic and applied microbiology*, 12, 2, 159–161

Schlesner, H., Rensmann, C., Tindall, B. J., Gade, D., Rabus, R., Pfeiffer, S., et al. (2004), Taxonomic heterogeneity within the planctomycetales as derived by dna–dna hybridization, description of rhodopirellula baltica gen. nov., sp. nov., transfer of pirellula marina to the genus blastopirellula gen. nov. as blastopirellula marina comb. nov. and emended description of the genus pirellula, *International journal of systematic and evolutionary microbiology*, 54, 5, 1567–1580

Shieh, W. Y., Liu, T. Y., Lin, S. Y., Jean, W. D., and Chen, J.-S. (2008), Simiduia agarivorans gen. nov., sp. nov., a marine, agarolytic bacterium isolated from shallow coastal water from keelung, taiwan, *International journal of systematic and evolutionary microbiology*, 58, 4, 895–900

Sikorski, J., Tindall, B. J., Lowry, S., Lucas, S., Nolan, M., Copeland, A., et al. (2010), Complete genome sequence of meiothermus silvanus type strain (vi-r2t), *Standards in genomic sciences*, 3, 1, 37

Sohn, J. H., Lee, J.-H., Yi, H., Chun, J., Bae, K. S., Ahn, T.-Y., et al. (2004), Kordia algicida gen. nov., sp. nov., an algicidal bacterium isolated from red tide, *International journal of systematic and evolutionary microbiology*, 54, 3, 675–680

Stackebrandt, E., Päuker, O., Steiner, U., Schumann, P., Sträubler, B., Heibei, S., et al. (2007), Taxonomic characterization of members of the genus corallococcus: molecular divergence versus phenotypic coherency., *Syst Appl Microbiol*, 30, 2, 109–118, doi:10.1016/j.syapm.2006.03.002

Suzuki, M., Nakagawa, Y., Harayama, S., and Yamamoto, S. (2001), Phylogenetic analysis and taxonomic study of marine cytophaga-like bacteria: proposal for tenacibaculum gen. nov. with tenacibaculum maritimum comb. nov. and tenacibaculum ovolyticum comb. nov., and description of tenacibaculum mesophilum sp. nov. and tenacibaculum amylolyticum sp. nov., *Int J Syst Evol Microbiol*, 51, Pt 5, 1639–1652

Tan, D., Xue, Y.-S., Aibaidula, G., and Chen, G.-Q. (2011), Unsterile and continuous production of polyhydroxybutyrate by halomonas td01., *Bioresour Technol*, 102, 17, 8130–8136, doi:10.1016/j.biortech.2011.05.068

Teh, B. S., Abdul Rahman, A. Y., Saito, J. A., Hou, S., and Alam, M. (2012), Complete genome sequence of the thermophilic bacterium thermus sp. strain ccb_us3_uf1, *Journal of bacteriology*, 194, 5, 1240

Thomas, F., Barbeyron, T., and Michel, G. (2011), Evaluation of reference genes for real-time quantitative pcr in the marine flavobacterium zobellia galactanivorans, *Journal of microbiological methods*, 84, 1, 61–66

Thompson, B. and Murray, R. (1981), Isolation and characterization of the plasma membrane and the outer membrane of deinococcus radiodurans strain sark, *Canadian journal of microbiology*, 27, 7, 729–734

Touchon, M., Barbier, P., Bernardet, J.-F., Loux, V., Vacherie, B., Barbe, V., et al. (2011), Complete genome sequence of the fish pathogen flavobacterium branchiophilum, *Applied and environmental microbiology*, 77, 21, 7656–7662

Triches, T. C., de Figueiredo, L. C., Feres, M., de Freitas, S. F. T., Zimmermann, G. S., and Cordeiro, M. M. R. (2014), Microbial profile of root canals of primary teeth with pulp necrosis and periradicular lesion., *J Dent Child (Chic)*, 81, 1, 14–19

Van Trappen, S., Vandecandelaere, I., Mergaert, J., and Swings, J. (2004a), Flavobacterium degerlachei sp. nov., flavobacterium frigoris sp. nov. and flavobacterium micromati sp. nov., novel psychrophilic bacteria isolated from microbial mats in antarctic lakes, *International journal of systematic and evolutionary microbiology*, 54, 1, 85–92

Van Trappen, S., Vandecandelaere, I., Mergaert, J., and Swings, J. (2004b), Gillisia limnaea gen. nov., sp. nov., a new member of the family flavobacteriaceae isolated from a microbial mat in lake fryxell, antarctica, *International journal of systematic and evolutionary microbiology*, 54, 2, 445–448

Vu, A. T., Nguyen, N. C., and Leadbetter, J. R. (2004), Iron reduction in the metal-rich guts of wood-feeding termites, *Geobiology*, 2, 4, 239–247

Wegner, C.-E., Richter, M., Richter-Heitmann, T., Klindworth, A., Frank, C. S., Glöckner, F. O., et al. (2014), Permanent draft genome of rhodopirellula sallentina sm41, *Marine genomics*, 13, 17–18

Wildgruber, G., Thomm, M., König, H., Ober, K., Richiuto, T., and Stetter, K. O. (1982), Methanoplanus limicola, a plate-shaped methanogen representing a novel family, the methanoplanaceae, *Archives of Microbiology*, 132, 1, 31–36

Wilharm, T., Zhilina, T., and Hummel, P. (1991), Dna-dna hybridization of methylotrophic halophilic methanogenic bacteria and transfer of methanococcus halophilusvp to the genus methanohalophilus as methanohalophilus halophilus comb. nov., *International journal of systematic bacteriology*, 41, 4, 558–562

Yoon, J., Maneerat, S., Kawai, F., and Yokota, A. (2006a), Myroides pelagicus sp. nov., isolated from seawater in thailand., *Int J Syst Evol Microbiol*, 56, Pt 8, 1917–1920, doi:10.1099/ijs.0.64336-0

Yoon, J.-H., Kang, S.-J., Lee, C.-H., and Oh, T.-K. (2006b), Donghaeana dokdonensis gen. nov., sp. nov., isolated from sea water., *Int J Syst Evol Microbiol*, 56, Pt 1, 187–191, doi:10.1099/ijs.0.63847-0
