# Supplementary material for: Domain organization of DNase from Thioalkalivibrio sp. provides insights into retention of activity in high salt environments
Source: Front Microbiol. 2015 Jul 1;6:661. doi: 10.3389/fmicb.2015.00661 (PMC4486849; doi:10.3389/fmicb.2015.00661)
Supplement: Supplementary file 4 [file Image1.PDF]

Supplementary Material: Domain organization of DNase from *Thioalkalivibrio sp.* suggests insights on how bacterial DNases can retain activity at extremes of ionic strength

Gediminas Alzbutas [1,2,*], **Milda Kaniusaite** [2], **Algirdas Grybauskas** [2,3] and **Arunas Lagunavicius** [2]

[1] *VU Institute of Biotechnology, V.A. Graiciuno 8, LT-02241 Vilnius, Lithuania*
[2] *Thermo Fisher Scientific, V.A. Graiciuno 8, LT-02241 Vilnius, Lithuania*
[3] *Vilnius University, Universiteto str. 3 LT-01513 Vilnius, Lithuania*

Correspondence*:
Gediminas Alzbutas
Thermo Fisher Scientific, V.A. Graiciuno 8, LT-02241 Vilnius, Lithuania,
gediminas.alzbutas@thermofisher.com

Extremophilic Industrially Important Enzymes and Molecular Mechanisms

1 SUPPLEMENTARY TABLES AND FIGURES

```
DNaseTA            ATGCTGCGCCTGGCAAGCTGGAACATCCAGCACCTGGGCTGGAACGTCGGTAAGGACTAC
DNaseTA H134A      ATGCTGCGCCTGGCAAGCTGGAACATCCAGCACCTGGGCTGGAACGTCGGTAAGGACTAC
DNaseTA ΔC         ATGCTGCGCCTGGCAAGCTGGAACATCCAGCACCTGGGCTGGAACGTCGGTAAGGACTAC

DNaseTA            CCGGCCGTCGCACGCATCGCGGCGCAGTTCGACTTTCTGGCGATCCAAGAAGTCATGAAC
DNaseTA H134A      CCGGCCGTCGCACGCATCGCGGCGCAGTTCGACTTTCTGGCGATCCAAGAAGTCATGAAC
DNaseTA ΔC         CCGGCCGTCGCACGCATCGCGGCGCAGTTCGACTTTCTGGCGATCCAAGAAGTCATGAAC

DNaseTA            GCCGAGGGTATTTACCGCCTGCGTGACACCCTGGAAGACGCGACCGGTGCCGAGTGGTCC
DNaseTA H134A      GCCGAGGGTATTTACCGCCTGCGTGACACCCTGGAAGACGCGACCGGTGCCGAGTGGTCC
DNaseTA ΔC         GCCGAGGGTATTTACCGCCTGCGTGACACCCTGGAAGACGCGACCGGTGCCGAGTGGTCC

DNaseTA            GTTCTGTACTCCGACGCGCTGGGCCGCAACACCTATCGTGAAAAGTACGCGTTCCTGTGG
DNaseTA H134A      GTTCTGTACTCCGACGCGCTGGGCCGCAACACCTATCGTGAAAAGTACGCGTTCCTGTGG
DNaseTA ΔC         GTTCTGTACTCCGACGCGCTGGGCCGCAACACCTATCGTGAAAAGTACGCGTTCCTGTGG

DNaseTA            CGTGAGGCCGCAGTTGAGTATGTCGGTGGTGCGCTGACCTACATCGACGAGGCCGACCGT
DNaseTA H134A      CGTGAGGCCGCAGTTGAGTATGTCGGTGGTGCGCTGACCTACATCGACGAGGCCGACCGT
DNaseTA ΔC         CGTGAGGCCGCAGTTGAGTATGTCGGTGGTGCGCTGACCTACATCGACGAGGCCGACCGT

DNaseTA            TTTGCCCGTGAGCCGTTCTCCGCGGTCTTTCGTTCCCGTGGCACGGATCAGCATTTCCTG
DNaseTA H134A      TTTGCCCGTGAGCCGTTCTCCGCGGTCTTTCGTTCCCGTGGCACGGATCAGCATTTCCTG
DNaseTA ΔC         TTTGCCCGTGAGCCGTTCTCCGCGGTCTTTCGTTCCCGTGGCACGGATCAGCATTTCCTG

DNaseTA            GCCGCCACGGTTCACATCACCTACGGCGACCGTGTTGCGGATCGTGTCGAGGAGATCGAG
DNaseTA H134A      GCCGCCACGGTTGCAATCACCTACGGCGACCGTGTTGCGGATCGTGTCGAGGAGATCGAG
DNaseTA ΔC         GCCGCCACGGTTCACATCACCTACGGCGACCGTGTTGCGGATCGTGTCGAGGAGATCGAG

DNaseTA            GCACTGCGTCGTTACTGGGACTGGCTGGCGGACGTCATGCCGGAGTACGCCGGCGAACGC
DNaseTA H134A      GCACTGCGTCGTTACTGGGACTGGCTGGCGGACGTCATGCCGGAGTACGCCGGCGAACGC
DNaseTA ΔC         GCACTGCGTCGTTACTGGGACTGGCTGGCGGACGTCATGCCGGAGTACGCCGGCGAACGC

DNaseTA            ATCCTGTTCGGCGACTTCAATCTGCCGCCGCACCACGACGGTTGGGCCTCGATGCGTGCG
DNaseTA H134A      ATCCTGTTCGGCGACTTCAATCTGCCGCCGCACCACGACGGTTGGGCCTCGATGCGTGCG
DNaseTA ΔC         ATCCTGTTCGGCGACTTCAATCTGCCGCCGCACCACGACGGTTGGGCCTCGATGCGTGCG

DNaseTA            GTTGCCGAACCGCTGGTCACCGAGGGCGCCACCACCCTGTCCACGCATGACCGTCGCTAC
DNaseTA H134A      GTTGCCGAACCGCTGGTCACCGAGGGCGCCACCACCCTGTCCACGCATGACCGTCGCTAC
DNaseTA ΔC         GTTGCCGAACCGCTGGTCACCGAGGGCGCCACCACCCTGTCCACGCATGACCGTCGCTAC

DNaseTA            GCGAATCTGTACGACAATCTGTGGGTTCCGAAGGATCACACGCTGCCGCTGGGTGATGCC
DNaseTA H134A      GCGAATCTGTACGACAATCTGTGGGTTCCGAAGGATCACACGCTGCCGCTGGGTGATGCC
DNaseTA ΔC         GCGAATCTGTACGACAATCTGTGGGTTCCGAAGGATCACACGCTGCCGCTGGGTGATGCC

DNaseTA            GGCATCCTGCCGTTCCCGGTCGTTCTGAGTGAGGTAACCGGTGTCTACTGGGATCACGAA
DNaseTA H134A      GGCATCCTGCCGTTCCCGGTCGTTCTGAGTGAGGTAACCGGTGTCTACTGGGATCACGAA
DNaseTA ΔC         GGCATCCTGCCGTTCCCGGTCGTTCTGAGTGAGGTAACCGGTGTCTACTGGGATCACGAA

DNaseTA            AAGGCCCGCGACCGTGTCTCCGACCATGCCCCGGTTTATGTACTGTTCGAAGGTAACACG
DNaseTA H134A      AAGGCCCGCGACCGTGTCTCCGACCATGCCCCGGTTTATGTACTGTTCGAAGGTAACACG
DNaseTA ΔC         AAGGCCCGCGACCGTGTCTCCGACCATGCCCCGGTTTATGTACTGTTCGAAGGT------

DNaseTA            CTGCACGACGCGGTTGTCGCGGAGATCGCCGATCAAGAAGCGGGCTGCATCGATCTGAAT
DNaseTA H134A      CTGCACGACGCGGTTGTCGCGGAGATCGCCGATCAAGAAGCGGGCTGCATCGATCTGAAT
DNaseTA ΔC         ------------------------------------------------------------

DNaseTA            CGCGCCTCGGTAAGCGAACTGACCGCCCTGCCGCATATTGGCGAGGCACGTGCGGAGGCG
DNaseTA H134A      CGCGCCTCGGTAAGCGAACTGACCGCCCTGCCGCATATTGGCGAGGCACGTGCGGAGGCG
DNaseTA ΔC         ------------------------------------------------------------

DNaseTA            ATCAAGGACGGTCGTCCGTGGAATGCCGTTCGTGACCTGAAAGAGATTCGCGGTATCGGT
DNaseTA H134A      ATCAAGGACGGTCGTCCGTGGAATGCCGTTCGTGACCTGAAAGAGATTCGCGGTATCGGT
DNaseTA ΔC         ------------------------------------------------------------

DNaseTA            GCTGGTCGTCTGGAGGAGATCAAGGCGCGTGGTGAGGCCTGCATCGAGCCGGGCCATCAC
DNaseTA H134A      GCTGGTCGTCTGGAGGAGATCAAGGCGCGTGGTGAGGCCTGCATCGAGCCGGGCCATCAC
DNaseTA ΔC         ---------------------------------------------------GGCCATCAC

DNaseTA            CATCACCACCACGGCTAA
DNaseTA H134A      CATCACCACCACGGCTAA
DNaseTA ΔC         CATCACCACCACGGCTAA
```

Supplementary Figure S1. Sequence alignment of DNaseTA and its mutants which properties were experimentally analysed.
