# Supplementary material for: Domain organization of DNase from Thioalkalivibrio sp. provides insights into retention of activity in high salt environments
Source: Front Microbiol. 2015 Jul 1;6:661. doi: 10.3389/fmicb.2015.00661 (PMC4486849; doi:10.3389/fmicb.2015.00661)
Supplement: Supplementary file 5 [file Image2.PDF]

Supplementary Material: Domain organization of DNase from *Thioalkalivibrio sp.* suggests insights on how bacterial DNases can retain activity at extremes of ionic strength

Gediminas Alzbutas [1,2,*], **Milda Kaniusaite** [2], **Algirdas Grybauskas** [2,3] **and Arunas Lagunavicius** [2]

[1] *VU Institute of Biotechnology, V.A. Graiciuno 8, LT-02241 Vilnius, Lithuania*
[2] *Thermo Fisher Scientific, V.A. Graiciuno 8, LT-02241 Vilnius, Lithuania*
[3] *Vilnius University, Universiteto str. 3 LT-01513 Vilnius, Lithuania*

Correspondence*:
Gediminas Alzbutas
Thermo Fisher Scientific, V.A. Graiciuno 8, LT-02241 Vilnius, Lithuania,
gediminas.alzbutas@thermofisher.com

Extremophilic Industrially Important Enzymes and Molecular Mechanisms

1 SUPPLEMENTARY TABLES AND FIGURES

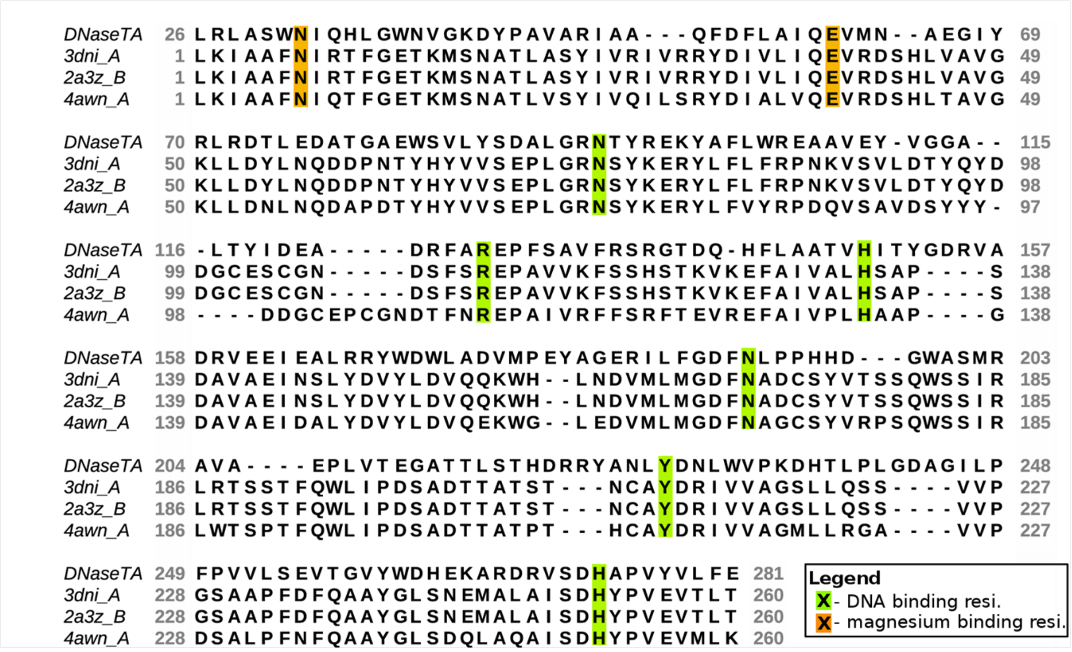

Supplementary Figure S2. Structural sequence alignment of DNase domains corresponding to DNaseTA (homology model), bovine (PDB IDs: 3dni, 2a3z) and human (PDB ID: 4awn) DNaseI. Colours indicate magnesium and DNA contacting residues in the eukaryotic proteins
